# Zbtb20 promotes astrocytogenesis during neocortical development

**DOI:** 10.1038/ncomms11102

**Published:** 2016-03-22

**Authors:** Motoshi Nagao, Toru Ogata, Yasuhiro Sawada, Yukiko Gotoh

**Affiliations:** 1 Department of Rehabilitation for the Movement Functions, Research Institute, National Rehabilitation Center for Persons with Disabilities, 4-1 Namiki, Tokorozawa, Saitama 359-8555, Japan; 2 Graduate School of Pharmaceutical Sciences, The University of Tokyo, 7-3-1 Hongo, Bunkyo-ku, Tokyo 113-0033, Japan

## Abstract

Multipotent neural precursor cells (NPCs) generate astrocytes at late stages of mammalian neocortical development. Many signalling pathways that regulate astrocytogenesis directly induce the expression of GFAP, a marker of terminally differentiated astrocytes. However, astrocyte specification occurs before GFAP expression and essential factors for the specification step have remained elusive. Here we show that Zbtb20 regulates astrocyte specification in the mouse neocortex. Zbtb20 is highly expressed in late-stage NPCs and their astrocytic progeny. Overexpression and knockdown of Zbtb20 promote and suppress astrocytogenesis, respectively, although Zbtb20 does not directly activate the *Gfap* promoter. Astrocyte induction by Zbtb20 is suppressed by knockdown of Sox9 or NFIA. Furthermore, in the astrocyte lineage, Zbtb20 directly represses the expression of *Brn2*, which encodes a protein necessary for upper-layer neuron specification. Zbtb20 is thus a key determinant of astrocytogenesis, in which it collaborates with Sox9 and NFIA, and acts in part through direct repression of *Brn2* expression.

## Abstract (web summary)

Astrocytes in the brain are derived from neural precursor cells (NPCs). Here, Motoshi Nagao and colleagues show that the transcription repressor Zbtb20 regulates astrocyte specification in the mouse neocortex.

## Graphical abstract

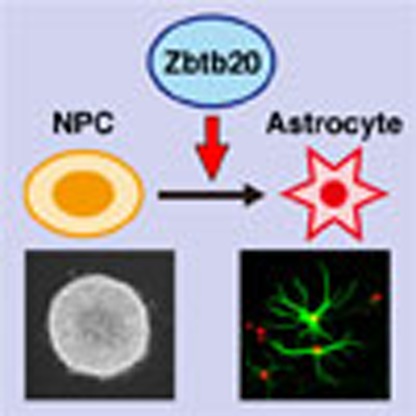

The mammalian neocortex is a complex and highly organized structure that contains diverse neuronal and glial cell types. In the developing central nervous system (CNS), neurons and two types of glial cells—astrocytes and oligodendrocytes—are generated from common multipotent neural precursor cells (NPCs)^1^. NPCs first give rise to neurons at early stages of brain development and subsequently differentiate into glia at later stages^2^,^3^. This precise temporal control of NPC fate is crucial for proper development of the CNS.

Astrocytes are the most numerous cell type in the mammalian brain and perform diverse functions such as recycling of neurotransmitters, energy storage, formation of the blood–brain barrier and regulation of synapse formation and function^4^,^5^,^6^. Astrocytogenesis begins towards the end of the neurogenic period and is regulated by extrinsic signals such as growth factors and cytokines, as well as by cell-intrinsic programmes such as epigenetic chromatin modification^2^,^3^,^4^,^5^,^6^,^7^,^8^. Members of the interleukin-6 family of cytokines—including leukemia inhibitory factor, ciliary neurotrophic factor (CNTF) and cardiotrophin-1—which activate the JAK-STAT (Janus kinase-signal transducer and activator of transcription) pathway have been shown to promote astrocyte differentiation^9^,^10^,^11^,^12^. In addition, the bone morphogenetic protein (BMP)–Smad and Notch signalling pathways engage in cross-talk with the JAK-STAT pathway and promote astrocyte differentiation^13^,^14^,^15^.

Previous studies of astrocytogenesis have relied primarily on the induction of glial fibrillary acidic protein (GFAP) expression as a marker of terminal astrocyte differentiation^4^,^5^,^6^,^7^,^8^ and have shown that the JAK-STAT and Smad pathways directly regulate the promoter of the *GFAP* gene^10^,^11^,^15^. Given that GFAP expression is also induced in reactive astrocytes during the response to injury^6^, the expression level of GFAP *per se* may not necessarily reflect the specification or differentiation of astrocytes. Furthermore, during the development of the CNS, the specification of astrocytic fate in NPCs occurs before GFAP induction^6^. It is therefore likely to be that astrocyte specification is determined by mechanisms other than the signalling pathways that regulate GFAP expression.

The transcription factors Sox9 and nuclear factor I/A (NFIA) are implicated in astrocyte specification in the embryonic spinal cord^16^,^17^,^18^,^19^. However, these molecules are also expressed in cells of the oligodendrocyte lineage^17^,^19^,^20^. Conditional knockout of Sox9 in the developing spinal cord leads to defects in the specification of both astrocytes and oligodendrocytes^19^. NFIA is expressed in oligodendrocyte precursor cells (OPCs) and antagonizes the ability of Sox10 to induce myelin genes, with the level of NFIA being downregulated before myelin gene expression^17^. Thus, Sox9 and NFIA do not exclusively mark precursors committed to the astrocytic fate. We therefore hypothesized that some other molecule or mechanism essential for astrocyte specification remains to be discovered.

Zinc finger- and BTB domain-containing protein 20 (Zbtb20) is a member of the BTB/POZ family of transcription factors and functions as a transcriptional repressor^21^,^22^. Zbtb20 is expressed in developing hippocampal neurons and plays a key role in hippocampal development and function^21^,^23^,^24^,^25^,^26^,^27^,^28^. Although Zbtb20 is expressed in astrocytes in the cerebral cortex and cerebellum^21^, its role in astrocyte development has not been elucidated. We have now identified Zbtb20 as an essential regulator of astrocyte development in the developing mouse CNS. We found that Zbtb20 is highly expressed in NPCs at late stages (during the gliogenic period) of neocortical development, as well as in differentiating and mature astrocytes. Overexpression and knockdown experiments *in vitro* and *in vivo* revealed that Zbtb20 promotes the production of astrocytes and suppresses that of neurons. Given that knockdown of Sox9 or NFIA attenuated the promotion of astrocyte production by Zbtb20, Zbtb20 appears to cooperate with Sox9 and NFIA during astrocyte development. Moreover, Zbtb20 directly repressed expression of the mouse brain-2 (*Brn2*) gene, with Brn2 being essential for neocortical neurogenesis. Our results suggest that Zbtb20, together with Sox9 and NFIA, promotes astrocytogenesis in part through the repression of *Brn2* expression in the neocortex.

## Results

### Zbtb20 expression closely correlates with astrocyte development

**Figure 1:**
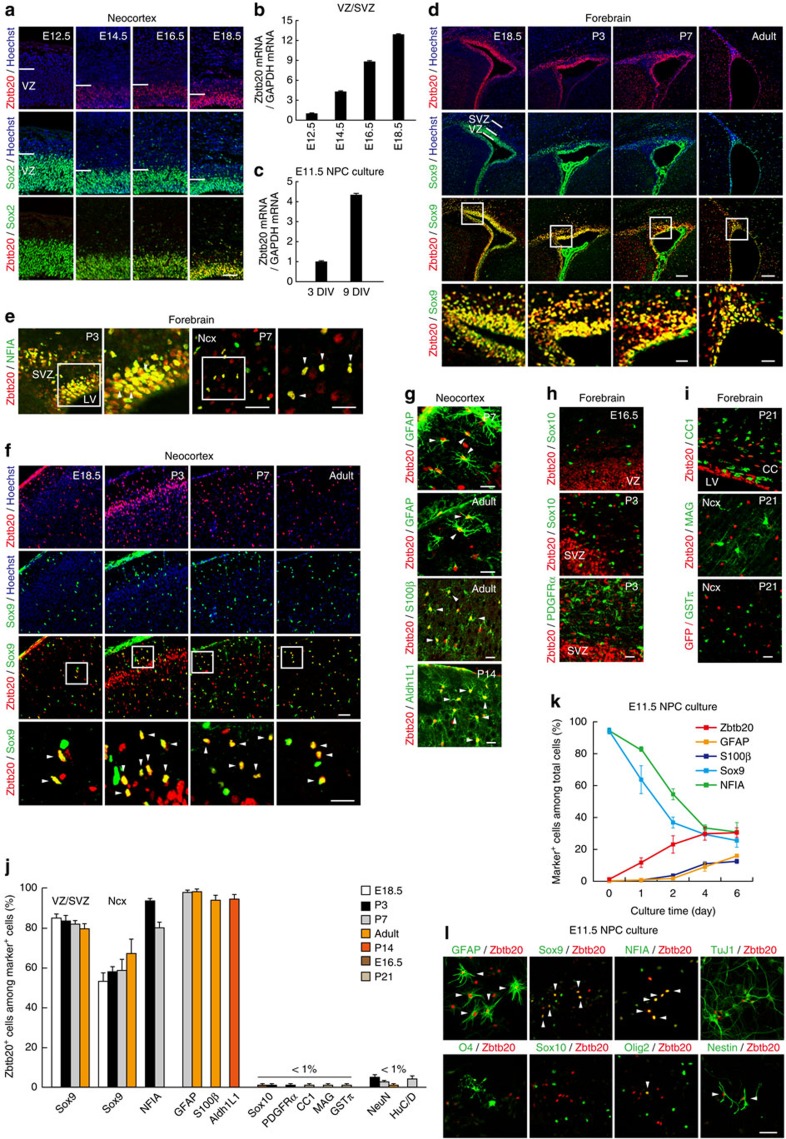
Expression of Zbtb20 in mouse brain and in cultured NPCs. (**a**) Immunofluorescnce staining of Zbtb20 and Sox2 in the embryonic neocortex. (**b**,**c**) Quantitative RT–PCR analysis of relative Zbtb20 mRNA abundance in the VZ/SVZ of the embryonic forebrain (**b**) and in NPCs cultured with FGF2 and EGF for 3 or 9 DIV (**c**). Data are means±s.d. (*n*=3). (**d**–**f**) Expression of Zbtb20, Sox9 and NFIA in the VZ/SVZ (**d**,**e**) and neocortex (**e**,**f**) at the indicated developmental stages. The bottom-most panels (**d**,**f**) or right panels of each pair (**e**) are higher magnification views of the boxed areas. (**g**) Double staining for Zbtb20 and either GFAP, S100β or Aldh1L1-GFP in the neocortex. (**h**,**i**) Double staining for Zbtb20 and either Sox10 or platelet-derived growth factor receptor-α (PDGFRα) (**h**), or for Zbtb20 and either CC1, myelin-associated glycoprotein (MAG) or glutathione *S*-transferase π (GSTπ) (**i**) in the forebrain. (**j**) Quantification of the percentages of Zbtb20^+^ cells among marker^+^ cells in **d**–**i** and Supplementary Fig. 3. Data are means±s.d. (*n*=3). (**k**) Time course of the emergence of Zbtb20^+^, Sox9^+^ or NFIA^+^ cells and of astrocytes after the induction of NPC differentiation in cultures. Data represent the percentages of marker^+^ cells among total cells and are means±s.d. (*n*=3). (**l**) NPCs cultured without FGF2 and EGF for 6 days were stained for the indicated markers. CC, corpus callosum; LV, lateral ventricle; Ncx, neocortex; SVZ, subventricular zone; VZ, ventricular zone. Arrowheads indicate double-positive cells (**e**–**g**,**l**). Scale bars, 50 μm (**a**,**e**,**f**,**l**), 100 μm (**d**) and 25 μm (**g**–**i** and higher magnification views in **d**–**f**).

We first examined the expression pattern of Zbtb20 in the developing mouse neocortex. Zbtb20 protein was first detected around embryonic day (E) 14.5 in the ventricular zone (VZ) of the cortex, where Sox2^+^ NPCs reside (Fig. 1a). The expression level increased gradually in the VZ and subventricular zone (SVZ) as development proceeded and Zbtb20 was highly expressed in late-stage NPCs, which preferentially generate glia (Fig. 1a,b). Neocortical NPCs isolated at E11.5 and cultured for 3 or 9 days *in vitro* (DIV) correspond to neurogenic and gliogenic stages, respectively^29^, and we found that the abundance of Zbtb20 messenger RNA was higher in 9 DIV cultures than in 3 DIV cultures (Fig. 1c). Given that these results suggested that the expression of Zbtb20 is associated with gliogenesis, we further examined such expression in the glial lineage at later stages. Many Zbtb20^+^ cells among NPCs and glial lineage cells in the VZ/SVZ and neocortex of the perinatal and adult brain coexpressed Sox9 or NFIA^16^,^17^,^18^,^19^,^30^,^31^ (Fig. 1d–f,j and Supplementary Fig. 1). Furthermore, most GFAP^+^ or S100β^+^ astrocytes expressed Zbtb20 in the neocortex (Fig. 1g,j). Analysis of the transgenic reporter line Aldh1L1-GFP, which expresses green fluorescent protein (GFP) under the control of the *Aldh1L1* gene promoter as an astrocyte marker, also revealed that Zbtb20 was expressed by most Aldh1L1-GFP^+^ cells (Fig. 1g,j). In contrast, Zbtb20 expression was not detected in cells positive for Sox10, a marker of the oligodendrocyte lineage, or in OPCs positive for platelet-derived growth factor receptor-α (Fig. 1h,j). Zbtb20 was also not expressed in mature oligodendrocytes positive for CC1, myelin-associated glycoprotein or glutathione *S*-transferase π (Fig. 1i,j). These results suggested that Zbtb20 is expressed in astrocytes but not in oligodendrocytes. In addition to cells of the astrocyte lineage, we found that Zbtb20 was expressed in S100β^+^ or FoxJ1^+^ ependymal cells^32^,^33^ in the postnatal and adult brains (Supplementary Fig. 2a) and in a small fraction of NeuN^+^ or HuC/D^+^ neurons at perinatal stages (Fig. 1j and Supplementary Fig. 3a,b). However, Zbtb20 was not expressed in NeuN^+^ neurons in the adult neocortex (Fig. 1j and Supplementary Fig. 3a), suggesting that the expression of Zbtb20 in the neuronal cells is transient.

We next examined the expression of Zbtb20 in NPC cultures derived from the E11.5 forebrain. Culture of the cells for 6 days in the absence of growth factors to induce differentiation revealed that a substantial population of Zbtb20^+^ cells began to emerge at day 1, before most GFAP^+^ or S100β^+^ astrocytes appeared at day 4, indicating that the expression of Zbtb20 precedes the induction of GFAP or S100β expression (Fig. 1k). At day 6, most Zbtb20^+^ cells (54.7%) were GFAP^+^ astrocytes, with a small fraction of Zbtb20^+^ cells (9.6%) being TuJ1^+^ neurons (marker^+^ cells/Zbtb20^+^ cells: Sox9, 82.2%; NFIA, 79.2%; O4, 0%; Sox10, 0%; Olig2, 3.4%; and nestin, 19.8%) (Fig. 1l). Consistent with the *in vivo* expression pattern, Zbtb20 was thus expressed in cells of the astrocyte lineage and a small fraction of neurons, but not in cells of the oligodendrocyte lineage, in *in vitro* cultures (Fig. 1k,l).

### Zbtb20 regulates astrocytogenesis from NPCs

**Figure 2:**
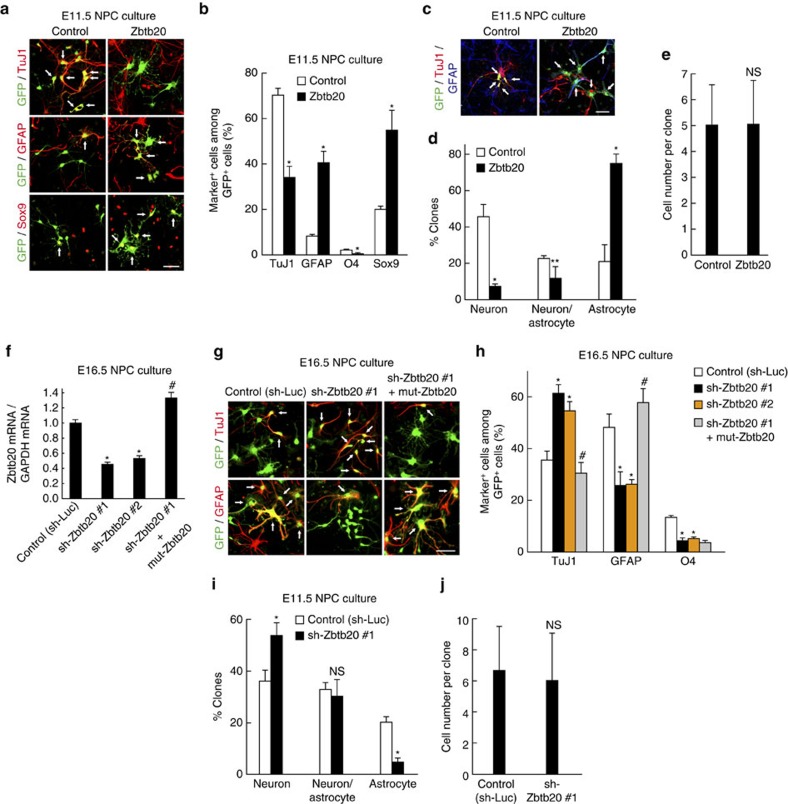
Zbtb20 is necessary and sufficient for astrocytogenesis of NPCs *in vitro*. (**a**,**b**) E11.5 NPCs were infected with retroviruses encoding GFP alone (control) or GFP plus Zbtb20 and were then cultured without FGF2 and EGF for 6 days before immunostaining for TuJ1, GFAP, O4, Sox9 and GFP (**a**). The percentages of marker^+^ cells among total GFP^+^ cells were quantified as means±s.d. (*n*=3 to 5) (**b**). (**c**–**e**) The control or Zbtb20-overexpressing cells were plated at a clonal density and cultured with FGF2 for 3 days and without FGF2 for 4 days, after which the percentages of clones containing only TuJ1^+^ cells (neuron-only clones), both TuJ1^+^ cells and GFAP^+^ cells (neuron- and astrocyte-containing clones) or only GFAP^+^ cells (astrocyte-only clones) among total clones were quantified (**c**,**d**). The cell number per clone was also determined (**e**). Data are means±s.d. (*n*=3). (**f**–**h**) E16.5 NPCs were infected with retroviruses encoding GFP together with either a control shRNA (sh-Luc), a Zbtb20 shRNA (sh-Zbtb20 #1 or #2), or sh-Zbtb20 #1 and an shRNA-resistant mutant of Zbtb20 (mut-Zbtb20). Knockdown efficiency of the shRNAs was determined by quantitative RT–PCR analysis; data are expressed relative to the control value and are means±s.d. (*n*=3) (**f**). The Zbtb20 shRNA-expressing NPCs were induced to differentiate for 6 days, after which the cells were immunostained for TuJ1, GFAP, O4 and GFP (**g**). The percentages of marker^+^ cells among total GFP^+^ cells were quantified; data are means±s.d. (*n*=3 to 5) (**h**). (**i**,**j**) The control or Zbtb20 knockdown cells were plated at a clonal density and then analysed as in **d** (**i**). The cell number per clone was also determined (**j**). Data are means±s.d. (*n*=3). Arrows indicate marker^+^/GFP^+^ cells (**a**,**c**,**g**). *P<0.01 and **P<0.05 versus corresponding control value; ^#^*P*<0.01 versus sh-Zbtb20 #1 value; NS, nonsignificant; *P*=0.98 (**e**), 0.55 (**i**) and 0.80 (**j**) versus corresponding control value. Scale bars, 50 μm.

Given that we found that Zbtb20 is highly expressed in late-stage NPCs and differentiated cells of the astrocyte lineage in the neocortex, we next asked whether Zbtb20 regulates astrocytogenesis. NPCs isolated from the E11.5 mouse forebrain were infected with retroviruses encoding GFP alone (control) or GFP together with Zbtb20, and they were then induced to differentiate for 6 days in the absence of growth factors. Compared with the control, overexpression of Zbtb20 increased the percentages of GFAP^+^ astrocytes and Sox9^+^ cells, and reduced those of both TuJ1^+^ neurons and O4^+^ oligodendrocytes (Fig. 2a,b). Similar results were obtained with NPCs isolated at E16.5, which have a higher gliogenic potential compared with E11.5 NPCs (Supplementary Fig. 4a,b). Furthermore, overexpression of Zbtb20 did not affect the proliferation or survival of NPCs, as assessed by 5-ethynyl-2′-deoxyuridine (EdU) incorporation and immunostaining of cleaved caspase 3, respectively (Supplementary Fig. 4c–e). The observed effects of Zbtb20 on NPC differentiation propensity therefore do not appear to be due to selective expansion or elimination of specific cell types. To determine whether the effects of Zbtb20 on astrocytogenesis are attributable either to the promotion of astrocyte fate commitment or to the selective proliferation of progenitors already committed to the astrocytic fate, we performed a clonal assay. Overexpression of Zbtb20 increased the percentage of astrocyte-only clones and reduced that of neuron-only clones (Fig. 2c,d), whereas it did not affect the size of the clones (Fig. 2e). In a clonal assay with E16.5 NPCs, overexpression of Zbtb20 had similar effects on NPC fate determination (Supplementary Fig. 5a,b,e). Furthermore, Zbtb20 overexpression reduced the percentage of oligodendrocyte-containing clones (Supplementary Fig. 5a,c,d). These results suggested that Zbtb20 promotes specification of multipotent NPCs into the astrocyte lineage.

We next investigated whether Zbtb20 is required for astrocytogenesis by knockdown of Zbtb20 with two different short hairpin RNAs (shRNAs) (Fig. 2f). Knockdown of Zbtb20 significantly enhanced the differentiation of NPCs into neurons at the expense of their differentiation into astrocytes (Fig. 2g,h). Expression of an shRNA-resistant mutant of Zbtb20 (mut-Zbtb20) (Fig. 2f) reversed the negative and positive effects of Zbtb20 knockdown on astrocytic and neuronal differentiation, respectively (Fig. 2g,h), indicating that the observed phenotypes of cells expressing the Zbtb20 shRNAs are not due to off-target effects. Furthermore, knockdown of Zbtb20 increased the percentage of neuron-only clones and reduced that of astrocyte-only clones without affecting the size of the clones (Fig. 2i,j). Similar results were observed in E16.5 NPC cultures (Supplementary Fig. 5f,i). These results suggested that Zbtb20 is required for astrocytogenesis of NPCs. Knockdown of Zbtb20 reduced the percentage of oligodendrocytes or oligodendrocyte-containing clones as was observed with the overexpression of Zbtb20 (Fig. 2b,h and Supplementary Fig. 5c,d,g,h). These may suggest that Zbtb20 is required for the generation of bipotent progenitors for astrocytes and oligodendrocytes, although it suppresses the subsequent production of OPCs.

**Figure 3:**
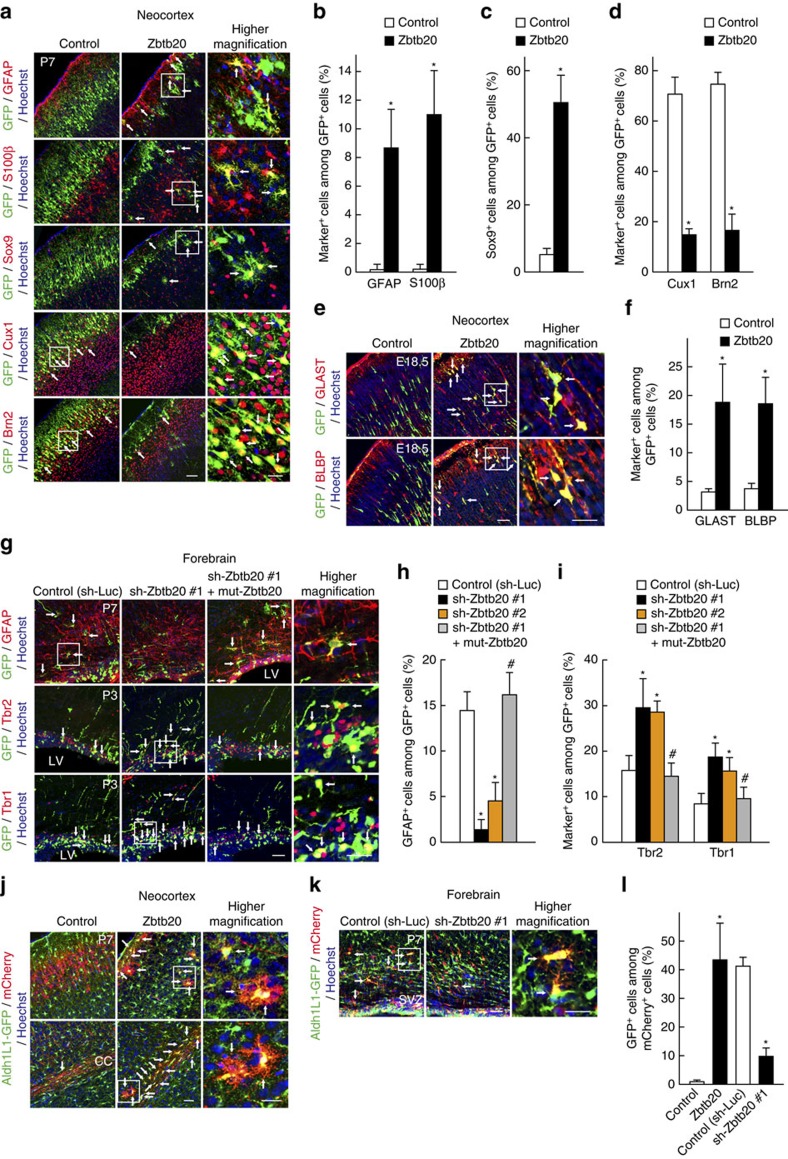
Zbtb20 is essential for astrocytogenesis *in vivo*. (**a**–**f**) Plasmids for GFP alone (control) or for both GFP and Zbtb20 were injected into the lateral ventricle of the E15.5 mouse forebrain *in utero* and were introduced into the dorsolateral region of the neocortex by electroporation. The brain was isolated at P7 or E18.5 and subjected to immunohistofluorescence analysis with antibodies to GFAP, to S100β, to Sox9, to Cux1, to Brn2 and to GFP (**a**), and to GLAST, to BLBP and to GFP (**e**). The percentages of marker^+^ cells among total GFP^+^ cells were determined as means±s.d. (*n*=3 to 5) (**b**–**d**,**f**). (**g**–**i**) Plasmids encoding GFP together with either a control shRNA (sh-Luc), a Zbtb20 shRNA (sh-Zbtb20 #1 or #2) or sh-Zbtb20 #1 and an shRNA-resistant mutant of Zbtb20 (mut-Zbtb20) were electroporated into the P0 mouse neocortex. The brain was isolated at P3 or P7 and subjected to immunostaining for GFAP at P7, Tbr2 and Tbr1 at P3, and GFP (**g**). The percentages of marker^+^ cells among total GFP^+^ cells were determined as means±s.d. (*n*=4 to 7) (**h**,**i**). (**j**–**l**) Plasmids for mCherry alone (control) or for both mCherry and Zbtb20 were electroporated into the E15.5 Aldh1L1-GFP mouse forebrain (**j**) and plasmids encoding mCherry together with either a control shRNA (sh-Luc) or a sh-Zbtb20 #1 were electroporated into the P0 Aldh1L1-GFP mouse neocortex (**k**). The brain was isolated at P7 and subjected to immunostaining for GFP and mCherry. The percentages of GFP^+^ cells among total mCherry^+^ cells were determined as means±s.d. (*n*=5 to 7) (**l**). CC, corpus callosum; LV, lateral ventricle; SVZ, subventricular zone. The right-most panels are higher magnification views of the boxed areas (**a**,**e**,**g**,**j**,**k**). Arrows indicate double-positive cells (**a**,**e**,**g**,**j**,**k**). *P<0.01 versus corresponding control value; ^#^*P*<0.01 versus sh-Zbtb20 #1 value. Scale bars, 75 μm (**a**), 50 μm (**e**,**g**,**j**,**k**) and 25 μm (higher magnification views in **a**,**e**,**g**,**j**,**k**).

To examine whether Zbtb20 plays a similar role in NPC differentiation *in vivo*, we performed *in utero* electroporation of mouse embryos at E15.5 with plasmids encoding either GFP alone (control) or both GFP and Zbtb20, and then analysed electroporated (GFP^+^) cells at postnatal day (P) 7. In the control neocortex, most GFP^+^ cells differentiated into neurons positive for Cux1, a marker of upper-layer neurons, with only a small percentage of GFP^+^ cells becoming GFAP^+^ or S100β^+^ astrocytes (Fig. 3a,b,d). Overexpression of Zbtb20 increased the percentage of GFAP^+^ or S100β^+^ astrocytes, as well as that of Sox9^+^ cells in the neocortex (Fig. 3a–c), whereas it reduced that of Cux1^+^ neurons (Fig. 3a,d). We also performed *in utero* electroporation in Aldh1L1-GFP mice with plasmids encoding mCherry alone (control) or mCherry together with Zbtb20. Overexpression of Zbtb20 remarkably increased the percentage of Aldh1L1-GFP^+^ cells among mCherry^+^ cells (Fig. 3j,l). The number of Zbtb20-overexpressing cells in the upper layer of the cortex was smaller than that of control cells, because more Zbtb20-overexpressing cells were found in the VZ/SVZ and corpus callosum compared with control (Fig. 3a,j and Supplementary Fig. 6a). The accumulations of the Zbtb20-overexpressing cells in the VZ/SVZ were already observed at E18.5 (Supplementary Fig. 6b). Therefore, the reduced number of the Zbtb20-overexpressing cells in the neocortex does not appear to be due to a selective loss of the cells. To reveal whether Zbtb20 regulates the early steps of astrocytogenesis *in vivo*, we further analysed the electroporated cells at an earlier developmental stage. Overexpression of Zbtb20 increased the percentage of cells positive for GLAST and BLBP are markers for both astrocyte precursors and radial glia^34^,^35^, at E18.5 (Fig. 3e,f). Although we observed Zbtb20 expression in ependymal cells, overexpression of Zbtb20 did not affect the production of ependymal cells (Supplementary Fig. 2a–c). Together, these results suggested that Zbtb20 also promotes astrocytogenesis *in vivo*.

Conversely, knockdown of Zbtb20 *in vivo* remarkably reduced the percentage of GFAP^+^ astrocytes in the neocortex and increased that of immature neurons or neuronal progenitors positive for the marker Tbr2 and that of neurons positive for the marker Tbr1 in the SVZ (Fig. 3g–i). Expression of mut-Zbtb20 prevented these effects of Zbtb20 knockdown (Fig. 3g–i). Knockdown of Zbtb20 also reduced the percentage of Aldh1L1-GFP^+^ astrocytes (Fig. 3k,l). These results thus suggested that Zbtb20 is indeed required for astrocytogenesis *in vivo*.

### Sox9 and NFIA cooperate with Zbtb20 in astrocytogenesis

**Figure 4:**
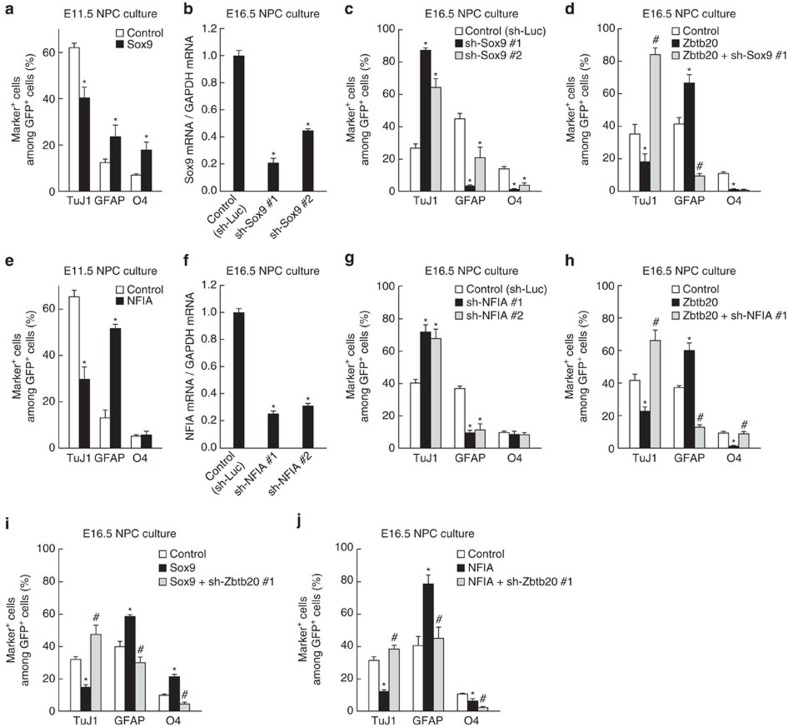
Zbtb20 regulates astrocyte differentiation of NPCs in a Sox9- and NFIA-dependent manner. (**a**,**e**) E11.5 NPCs were infected with retroviruses encoding GFP alone (control) or GFP plus either Sox9 (**a**) or NFIA (**e**) and were then cultured without FGF2 and EGF for 6 days before immunostaining for TuJ1, GFAP, O4 and GFP. The percentages of marker^+^ cells among total GFP^+^ cells were quantified as means±s.d. (*n*=3). (**b**,**c**,**f**,**g**) E16.5 NPCs were infected with retroviruses encoding GFP together with either a control shRNA (sh-Luc) or a Sox9 (**b**,**c**), or NFIA (**f**,**g**) shRNA (#1 or #2). Knockdown efficiency of the shRNAs was determined by quantitative RT–PCR analysis; data are expressed relative to the control value and are means±s.d. (*n*=3) (**b**,**f**). The cells were also induced to differentiate for 6 days and then analysed as in **a** and **e**; data are means±s.d. (*n*=3) (**c**,**g**). (**d**,**h**) E16.5 NPCs were infected with retroviruses for control, Zbtb20 either alone or together with sh-Sox9 #1 (**d**) or sh-NFIA #1 (**h**), induced to differentiate for 6 days, and then analysed for differentiation markers as in **a** and **e**; data are means±s.d. (*n*=3). (**i**,**j**) E16.5 NPCs were infected with retroviruses for control, Sox9 either alone or together with sh-Zbtb20 #1 (**i**) or control, NFIA either alone or together with sh-Zbtb20 #1 (**j**), induced to differentiate for 6 days, and then analysed for differentiation markers as in **a** and **e**; data are means±s.d. (*n*=3). *P<0.01 versus corresponding control value; ^#^*P*<0.01 versus value for Zbtb20 (**d**,**h**), Sox9 (**i**) or NFIA (**j**).

**Figure 5:**
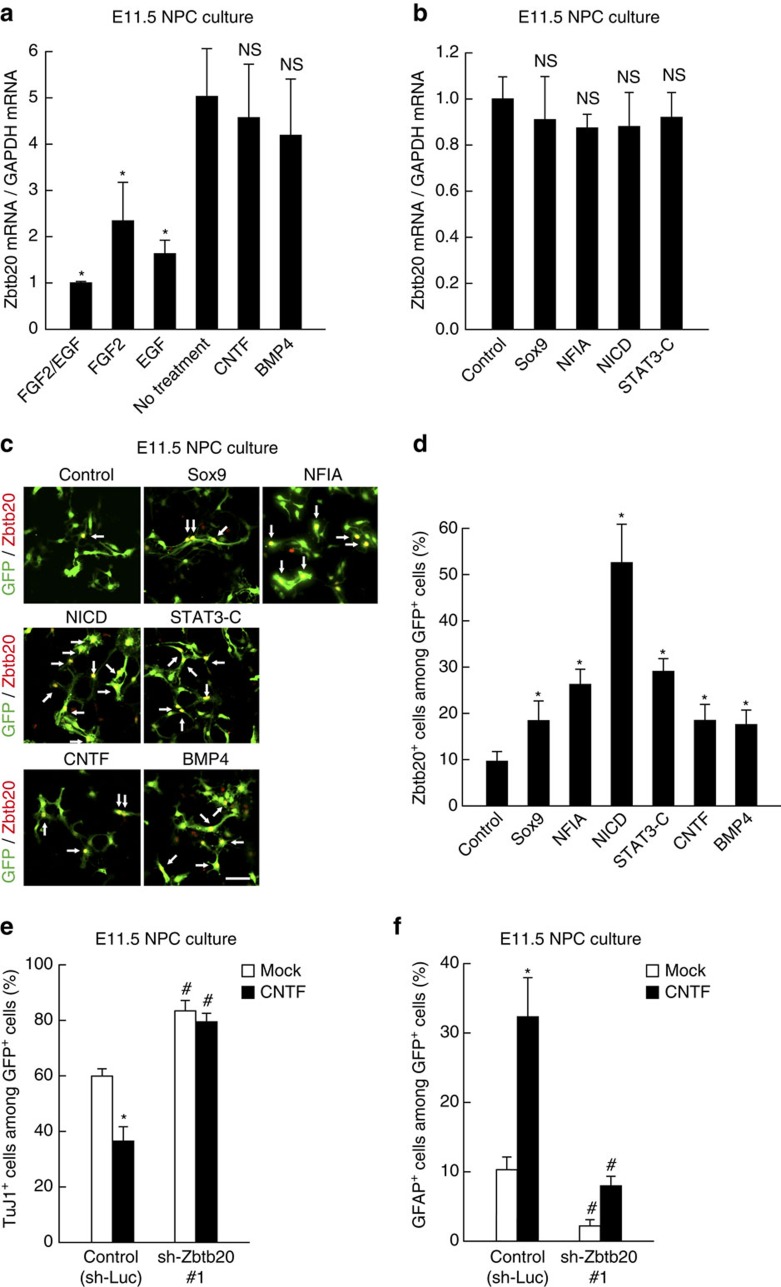
Regulation of Zbtb20 expression by gliogenic factors and effect of Zbtb20 knockdown on astrocyte differentiation induced by CNTF. (**a**,**b**) E11.5 NPCs were cultured without (No treatment) or with the indicated factors for 1 day (**a**) or they were infected with control, Sox9, NFIA, NICD or STAT3-C retroviruses and then cultured under differentiation-inducing conditions for 1day (**b**). The relative abundance of Zbtb20 mRNA was then measured by quantitative RT–PCR analysis. Data are means±s.d. (*n*=3). (**c**,**d**) E11.5 NPCs were infected with control, Sox9, NFIA, NICD or STAT3-C retroviruses and then cultured without FGF2 and EGF for 1 day. Control virus-infected cells were also treated with CNTF or BMP4 for 1 day. All cells were then subjected to immunostaining for Zbtb20 and GFP (**c**). Arrows indicate GFP^+^/Zbtb20^+^ cells. Scale bar, 50 μm. The percentages of Zbtb20^+^ cells among total GFP^+^ cells were quantified as means±s.d. (*n*=3) (**d**). (**e**,**f**) E11.5 NPCs were infected with control (sh-Luc) or Zbtb20 shRNA retroviruses, cultured without (Mock) or with CNTF for 6 days and then stained for TuJ1 (**e**), GFAP (**f**) and GFP for determination of the percentages of marker^+^ cells among total GFP^+^ cells (means±s.d., *n*=3). *P<0.01 versus value for No treatment (**a**), control virus-infected cells (**d**) or mock-treated cells (**e**,**f**); ^#^*P*<0.01 versus value for control virus-infected cells; NS, nonsignificant; *P*=0.63 (CNTF) and 0.41 (BMP4) versus value for No treatment (**a**); *P*=0.50 (Sox9), 0.09 (NFIA), 0.26 (NICD) and 0.40 (STAT3-C) versus control value (**b**).

**Figure 6:**
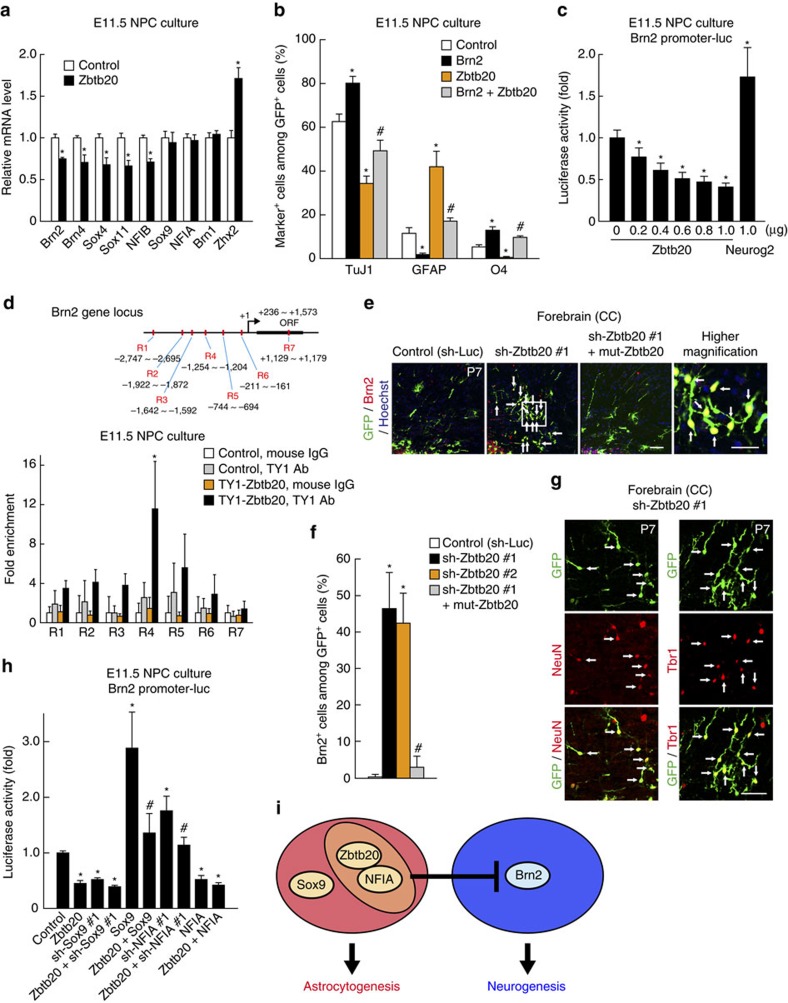
Zbtb20 inhibits *Brn2* gene expression. (**a**) Quantitative RT–PCR analysis of relative mRNA abundance for the indicated proteins in Zbtb20-overexpressing and control NPCs. Data are means±s.d. (*n*=3). (**b**) E11.5 NPCs were infected with retroviruses for control, Brn2, Zbtb20 or Brn2 plus Zbtb20 and induced to differentiate for 6 days. The percentages of marker^+^ cells among total GFP^+^ cells were determined as means±s.d. (*n*=4). (**c**) Luciferase assay of relative *Brn2* promoter activity and its concentration-dependent inhibition by Zbtb20 overexpression in NPCs. The effect of Neurog2 was examined as a positive control. Data are means±s.d. (*n*=5). (**d**) ChIP analysis of Zbtb20 binding to the *Brn2* promoter region in NPCs. Seven different regions (R1–R7) of the *Brn2* locus were tested in control cells and cells expressing TY1-tagged Zbtb20. Data are expressed as fold enrichment relative to the corresponding value for control cells and normal mouse immunoglobulin G (IgG). Data are means±s.d. (*n*=3 to 5). ORF, open reading frame. (**e**–**g**) Control, sh-Zbtb20 #1, sh-Zbtb20 #2 or sh-Zbtb20 #1 and mut-Zbtb20 plasmids were electroporated into P0 mouse neocortical NPCs. The P7 brain (CC, corpus callosum) was immunostained for Brn2 (**e**), NeuN, Tbr1 (**g**) and GFP. Arrows indicate marker^+^/GFP^+^ cells. The boxed region in **e** is shown at higher magnification in the right-most panel. Scale bars, 50 and 25 μm (higher magnification image in **e**). The percentages of Brn2^+^ cells among total GFP^+^ cells were determined as means±s.d. (*n*=5 to 7) (**f**). (**h**) Luciferase assay of relative *Brn2* promoter activity in NPCs transfected with plasmids for Zbtb20, Sox9, NFIA, sh-Sox9 #1 or sh-NFIA #1, as indicated. Data are means±s.d. (*n*=5). *P<0.01 and **P<0.05 versus the corresponding control value (**a**,**b**,**f**,**h**), 0 μg of Zbtb20 plasmid (**c**) or TY1-Zbtb20, mouse IgG (**d**); ^#^*P*<0.01 versus value for Zbtb20 (**b**,**h**) or sh-Zbtb20 #1 (**f**). (**i**) Model for astrocytogenesis by Zbtb20, Sox9 and NFIA. Zbtb20 and NFIA suppress *Brn2* expression.

Sox9 and NFIA are thought to play key roles in astrocyte specification and differentiation^16^,^17^,^18^,^19^,^31^,^36^,^37^. Therefore, we next asked whether the promotion of astrocytogenesis by Zbtb20 depends on Sox9 and NFIA in NPC cultures. Overexpression of Sox9 promoted both astrocyte and oligodendrocyte differentiation, whereas it suppressed neuronal differentiation (Fig. 4a). Overexpression of NFIA promoted astrocyte differentiation and suppressed neuronal differentiation (Fig. 4e). Knockdown of Sox9 or NFIA had opposite effects (compared with corresponding overexpression) on the differentiation propensity of NPCs (Fig. 4b,c,f,g). These results thus confirmed that Sox9 and NFIA are important factors for glial differentiation. Knockdown of either Sox9 or NFIA remarkably attenuated the promotion of astrocyte differentiation by Zbtb20 overexpression, as well as reversed the inhibitory effect of Zbtb20 on neuronal differentiation (Fig. 4d,h and Supplementary Fig. 7a,b). These results thus suggested that Sox9 and NFIA are required for the promotion of astrocyte differentiation by Zbtb20. Conversely, knockdown of Zbtb20 suppressed the promotion of astrocyte differentiation by either Sox9 or NFIA, suggesting that Zbtb20 is required for the induction of astrocytogenesis by Sox9 and NFIA (Fig. 4i,j). However, Zbtb20 does not appear to influence the level of Sox9 or NFIA gene transcription, given that overexpression of Zbtb20 did not increase the amounts of Sox9 and NFIA mRNAs (see Fig. 6a). Overexpression of Sox9 or NFIA also did not increase the abundance of Zbtb20 mRNA (see Fig. 5b), but it did slightly increase the percentage of Zbtb20-expressing cells (see Fig. 5c,d). Sox9 and NFIA may therefore act together or in parallel with Zbtb20 in astrocytogenesis, or they may render NPCs permissive to the induction of Zbtb20 expression in response to other (astrocyte-inducing) cues.

### Relation between Zbtb20 and astrocyte-regulating factors

We next asked what regulates Zbtb20 during astrocyte induction in the proper developmental context. We first examined the effects of the secreted factors CNTF and BMP, which promote astrocyte differentiation through the JAK-STAT and Smad pathways, respectively^9^,^10^,^11^,^12^,^13^,^14^,^15^. Treatment of NPCs with CNTF or BMP4 did not increase the level of Zbtb20 mRNA (Fig. 5a), although these factors did increase the percentage of NPCs expressing Zbtb20 (Fig. 5c,d). The Notch signalling pathway also promotes astrocyte differentiation^13^,^14^ but we found that forced activation of this pathway or of the STAT pathway by expression of the intracellular form of Notch1 (NICD) or an activated form of STAT3 (STAT3-C), respectively, also did not increase the abundance of Zbtb20 mRNA in NPCs (Fig. 5b). Again, however, expression of NICD and, to a lesser extent, that of STAT3-C increased the percentage of Zbtb20-expressing cells (Fig. 5c,d). These findings thus suggested that activation of Notch signalling (and perhaps JAK-STAT and Smad signalling) renders NPCs permissive for Zbtb20 expression during cell fate determination.

Conversely, it was also possible that Zbtb20 renders NPCs competent, to respond to extrinsic factor-mediated astrocyte differentiation. Consistent with previous observations^9^,^10^,^12^,^14^, CNTF promoted astrocyte differentiation and suppressed neuronal differentiation of NPCs (Fig. 5e,f). However, these effects of CNTF were significantly attenuated by knockdown of Zbtb20 (Fig. 5e,f), consistent with the notion that expression of Zbtb20 renders NPCs responsive to CNTF-mediated astrocyte differentiation. We have previously shown that Notch signalling pathway promotes astrocyte differentiation in the presence of CNTF^14^. Interestingly, however, knockdown of Zbtb20 did not affect the regulation of NPC differentiation by NICD alone or the promotion of astrocyte differentiation by NICD and CNTF (Supplementary Fig. 8). These results suggested that Notch signalling pathway can promote astrocyte differentiation independent of Zbtb20.

Fibroblast growth factor 2 (FGF2) and epidermal growth factor (EGF) are key factors that suppress the differentiation of NPCs and promote their maintenance (and proliferation). However, it has remained unclear how these factors suppress NPC differentiation, especially differentiation towards the astrocytic lineage. We found that FGF2 and EGF each remarkably reduced the abundance of Zbtb20 mRNA (Fig. 5a). Consistent with these results, only a small population of E11.5 NPCs (∼2%) expressed Zbtb20 in the presence of both FGF2 and EGF (day 0 in Fig. 1k). Given the suppression of astrocytogenesis in the absence of Zbtb20 (see above), the downregulation of Zbtb20 by FGF2 and EGF may explain, at least in part, the inhibition of astrocyte differentiation by these factors.

### Zbtb20 restricts *Brn2* expression in astrocyte lineage cells

Although Zbtb20 is necessary for CNTF-induced astrocyte differentiation (see above) and CNTF is known to activate the promoter of the *GFAP* gene through the JAK-STAT pathway^10^, Zbtb20 overexpression in NPCs did not increase the activity of this promoter (Supplementary Fig. 9). In contrast to the CNTF-JAK-STAT, BMP-Smad and Notch-NFIA pathways that directly regulate *GFAP* gene expression^10^,^15^,^31^,^37^, Zbtb20 therefore appears to promote astrocytogenesis by regulating target genes other than that for GFAP.

To identify the target genes of Zbtb20, we performed microarray analysis of Zbtb20-overexpressing NPCs. There was little difference in the expression of many astrocyte-related genes between control and Zbtb20-overexpressing NPCs, although significant increases in the abundance of GFAP and S100β mRNAs were observed in the Zbtb20-overexpressing cells (Supplementary Fig. 10a). The abundance of the mRNA for Zhx2, which contributes to the maintenance of NPCs^38^, was also found to be increased in Zbtb20-overexpressing cells by both microarray analysis (1.34-fold increase) and quantitative reverse transcriptase–PCR (RT–PCR) analysis (Fig. 6a). Given that Zbtb20 has been shown to act as a transcriptional repressor^22^, we examined whether Zbtb20 suppresses the expression of neuronal genes. Microarray analysis revealed that the expression level of certain neuronal genes^39^ was downregulated by Zbtb20 overexpression (Supplementary Fig. 10b). We validated these changes in expression level by quantitative RT–PCR analysis (Fig. 6a). Among these genes, we focused on that for Brn2 (also known as Pou3f2), which is an essential transcription factor in neocortical neurogenesis^40^,^41^,^42^,^43^. Overexpression of Brn2 enhanced neuronal differentiation and attenuated astrocyte differentiation in neocortical NPC cultures, and prevented the effects of Zbtb20 overexpression on NPC differentiation (Fig. 6b). We also found that overexpression of Zbtb20 significantly inhibited the activity of the *Brn2* gene promoter in NPCs (Fig. 6c). In addition, chromatin immunoprecipitation (ChIP) analysis revealed that Zbtb20 protein was highly enriched in a region of the *Brn2* promoter surrounding position –1,200 bp relative to the transcription start site in NPCs (Fig. 6d), suggesting that Zbtb20 directly binds to the promoter and thereby represses transcription. Furthermore, overexpression of Zbtb20 *in vivo* reduced the number of Brn2^+^ neurons (Fig. 3a,d). Conversely, Brn2^+^, NeuN^+^ and Tbr1^+^ neurons were ectopically induced by Zbtb20 knockdown, suggesting that Zbtb20 is necessary for suppression of *Brn2* expression *in vivo* (Fig. 6e–g). This knockdown phenotype was rescued by overexpression of mut-Zbtb20 (Fig. 6e,f). Together, these results suggested that Zbtb20 regulates the differentiation propensity of NPCs in part through the suppression of *Brn2* expression.

Given that Zbtb20 cooperates with Sox9 and NFIA in the induction of astrocytogenesis, we next asked whether Zbtb20 regulates the expression of *Brn2* in collaboration with Sox9 and NFIA. Overexpression of NFIA alone, as well as that of Zbtb20, suppressed *Brn2* promoter activity, and knockdown of NFIA, but not that of Sox9, relieved the repression of *Brn2* promoter activity induced by Zbtb20 overexpression (Fig. 6h). These results suggested that NFIA is required for suppression of *Brn2* expression by Zbtb20. Furthermore, co-immunoprecipitation experiments showed that Zbtb20 physically interacted with NFIA, but not Sox9 (Supplementary Fig. 11a,b). Together, these results suggested the possibility that Zbtb20 and NFIA form a complex and cooperatively repress *Brn2* expression (Fig. 6i).

## Discussion

Astrocyte development is regulated by extrinsic signals and cell-intrinsic programmes^2^,^3^,^4^,^5^,^6^,^7^,^8^, but our understanding of astrocytogenesis, especially its specification step, has lagged behind that of neurogenesis and oligodendrocyte development. One reason for this difference is that many studies of astrocytogenesis have relied primarily on measurement of the induction of GFAP expression, given that many factors associated with astrocyte development directly induce the expression of this protein^4^,^5^,^6^,^10^,^11^,^15^. However, the specification of astrocytes from NPCs occurs before GFAP induction during the development of the CNS^6^. Another reason is that specific markers for astrocyte precursors remain to be identified. We now provide evidence that Zbtb20 is a regulator of astrocytogenesis in the developing mouse neocortex. We found that the expression of Zbtb20 begins in the neocortical VZ around the mid-gestation stage and becomes high in late-stage NPCs and in astrocytes, but not in cells of the oligodendrocyte lineage, during neocortical development. Importantly, Zbtb20 expression is induced before GFAP expression during astrocytogenesis and Zbtb20 does not directly induce GFAP expression. Nevertheless, overexpression or knockdown of Zbtb20 *in vitro* or *in vivo* promoted and suppressed astrocytogenesis, respectively. Our findings thus suggest that Zbtb20 regulates the early steps of astrocytogenesis and defines astrocyte lineage cells in the neocortex.

We have shown that Zbtb20 is expressed in astrocyte lineage cells of the neocortex and regulates neocortical astrocytogenesis, but is Zbtb20 a region-specific factor for astrocytogenesis or does it also regulate astrocytogenesis in other parts of the CNS? To address the issue, we examined whether Zbtb20 is expressed in other regions of the CNS. Zbtb20 was expressed in Aldh1L1-GFP^+^, GFAP^+^ or S100β^+^ astrocytes in the ventral forebrain, the hippocampus and the grey and white matter of the spinal cord (Supplementary Fig. 12a–c). To further examine whether Zbtb20 promotes astrocytogenesis in the CNS regions other than neocortex, Zbtb20 was overexpressed in NPCs isolated from the E11.5 mouse spinal cord and the cells were induced to differentiate. In line with what we observed in neocortical NPCs, overexpression of Zbtb20 promoted astrocyte differentiation and suppressed neuronal and oligodendrocyte differentiation in the cultures of spinal cord NPCs (Supplementary Fig. 13a,b). These results suggest that Zbtb20 may be a pan-astrocytic factor that regulates astrocytogenesis in the broader regions of the CNS.

Our results show that Zbtb20 suppresses oligodendrocyte differentiation and neurogenesis in NPC cultures (Fig. 2b and Supplementary Fig. 4b); thus, how does Zbtb20 control oligodendrocyte development? Our microarray analysis showed that the expression level of Sox10, a transcription factor essential for oligodendrocyte differentiation^44^, was reduced (0.71-fold) in Zbtb20-overexpressing cells. We also validated this decrease in the expression level of Sox10 in Zbtb20-overexpressing cells using quantitative RT–PCR analysis (Supplementary Fig. 14a). In NPC cultures, overexpression of Zbtb20 remarkably reduced the percentage of Sox10^+^ cells immediately after the induction of differentiation (Supplementary Fig. 14b,c). To examine whether Zbtb20 suppresses the appearance of Sox10^+^ cells *in vivo*, we electroporated plasmids encoding either GFP alone (control) or both GFP and Zbtb20 into the lateral dorsoventral boundary of E15.5 mouse brains, which has been shown to be a source of cortical oligodendrocytes in the adult brain^45^, and then analysed electroporated cells at P3. Overexpression of Zbtb20 reduced the percentage of Sox10^+^ cells in the SVZ and corpus callosum compared with control (Supplementary Fig. 14d,e). These results suggest that Zbtb20 may directly repress *Sox10* expression and thereby suppress oligodendrocyte differentiation. To examine the possibility, we performed ChIP analysis in NPCs. We did not observe the enrichment of Zbtb20 in the *Sox10* gene region we examined, suggesting that Zbtb20 may regulate *Sox10* expression through indirect mechanisms. However, we cannot rule out the possibility that the downregulation of expression level of Sox10 or the reduction of Sox10^+^ cells observed in Zbtb20 overexpression is the consequence of a cell fate switch from oligodendrocytes to astrocytes.

Although several studies have demonstrated the existence of astrocyte precursors^46^,^47^,^48^, specific markers for these cells remain largely uncharacterized. Our results, together with previous observations showing Olig2 expression in both astrocyte and oligodendrocyte precursors^49^,^50^,^51^,^52^,^53^,^54^,^55^, suggest that astrocyte precursors or immature astrocytes are Zbtb20^+^/Sox9^+^/Olig2^+^ cells. This notion is supported by our finding that Zbtb20^+^/Olig2^+^ cells are negative for Sox10, a marker of OPCs (Supplementary Fig. 15a). The morphological features of Zbtb20^+^/Olig2^+^/Ki67^+^ cells (Supplementary Fig. 15b) coincide with those of proliferating non-radial glial cells (intermediate astrocyte precursors) that have recently been shown to serve to expand the local astrocyte population in the forebrain and spinal cord^47^,^48^.

In mice, the Zbtb family of proteins includes >40 members, several of which have been implicated in glial development. Zbtb45 is thus required for proper glial differentiation of NPCs^56^. In addition, PLZF (also known as Zbtb16) promotes NPC maintenance by increasing FGF receptor 3 expression and STAT3 activity, thereby suppressing neurogenesis and biasing NPCs towards gliogenesis in the spinal cord^57^. Given that Zbtb16 and Zbtb45 are similar to Zbtb20 in that these factors are involved in glial differentiation of NPCs, we examined whether Zbtb16 and Zbtb45 rescue the Zbtb20 knockdown phenotypes in NPC cultures. Overexpression of Zbtb16 or Zbtb45 failed to rescue the Zbtb20 knockdown phenotypes, preventing neither the promotion of neurogenesis nor the suppression of astrocytogenesis (Supplementary Fig. 16a–d). These results suggest that Zbtb20 has distinct functions from Zbtb16 and Zbtb45 in the regulation of gliogenesis.

In *Drosophila*, Glial cells missing acts as a master regulator of glial development and induces the expression of three transcription factors: Pointed (Pnt), Reversed polarity (Repo) and Tramtrack (Ttk)^58^. These factors regulate glial development by activating glial genes (*Pnt* and *Repo*) and suppressing neuronal genes (*Repo* and *Ttk*)^58^. Of note, given that, similar to Zbtb20, Ttk is a gliogenic factor with a BTB/POZ zinc finger domain, it may be a *Drosophila* homologue of Zbtb20. Our findings thus suggest that certain aspects of the molecular mechanisms underlying glial development are conserved between mammals and flies.

Astrocyte dysfunction has been implicated in neurodevelopmental diseases such as Rett syndrome and Alexander disease, neurodegenerative diseases such as Alzheimer's disease and amyotrophic lateral sclerosis, as well as psychiatric diseases such as major depressive disorder^4^,^59^. Hypermethylation within the coding region of the *Zbtb20* gene is associated with major depressive disorder^60^. Given that expression of Zbtb20 is maintained in mature astrocytes after astrocyte differentiation, its inactivation in astrocytes of the adult brain may contribute to the pathogenesis of this disorder. Further studies are thus warranted to determine the roles of Zbtb20 in astrocyte maturation and function in relation to pathological conditions of the developing and adult brain.

## Methods

### Animals

Pregnant ICR mice were obtained from CLEA Japan and Charles River Laboratories Japan. Aldh1L1-GFP mice were obtained from GENSAT. All mice were maintained and studied according to protocols approved by the Animal Care and Use Committees of the National Rehabilitation Center for Persons with Disabilities and The University of Tokyo.

### Antibodies

Immunostaining was performed with the following antibodies: Zbtb20 (Sigma-Aldrich, HPA016815, rabbit, 1:200 dilution), βIII-tubulin (TuJ1, Covance, MMS-435P, mouse, 1:5,000), GFAP (Millipore, MAB360, mouse, 1:1,000; Dako, Z0334, rabbit, 1:1,000; Abcam, ab4674, chicken, 1:2,000), S100β (Dako, Z0311, rabbit, 1:500; Sigma-Aldrich, SH-B1, mouse, 1:200), O4 (Millipore, MAB345, mouse, 1:1,000), Sox9 (Millipore, AB5535, rabbit, 1:1,000; Abcam, ab76997, mouse, 1:1,000; Santa Cruz Biotechnology, sc17342, goat, 1:100), Sox10 (Santa Cruz Biotechnology, sc17342, goat, 1:500), Sox2 (Santa Cruz Biotechnology, sc17320, goat, 1:200), Brn2 (Santa Cruz Biotechnology, sc6029, goat, 1:200), Olig2 (IBL, 18953, rabbit, 1:1,000; Millipore, AB9610, rabbit, 1:1,000; Millipore, MABN50, mouse, 1:500), NFIA (Sigma-Aldrich, HPA006111, rabbit, 1:100), Cux1 (Santa Cruz Biotechnology, sc13024, rabbit, 1:200), Tbr1 (Abcam, ab31940, rabbit, 1:2,000), Tbr2 (Abcam, ab23345, rabbit, 1:2,000), cleaved caspase 3 (Cell Signaling Technology, 9661, rabbit, 1:1,000), nestin (BD Biosciences, 556309, mouse, 1:200), NeuN (Millipore, MAB377, mouse, 1:200; Millipore, ABN78, rabbit, 1:1,000), HuC/D (Life Technologies, A21271, mouse, 1:200), CC1 (Millipore, OP80, mouse, 1:500), myelin-associated glycoprotein (Millipore, MAB1567, mouse, 1:400), glutathione *S*-transferase π (BD Biosciences, 610719, mouse, 1:1,000), platelet-derived growth factor receptor-α (BD Biosciences, 558774, rat, 1:200), Ki67 (Abcam, ab15580, rabbit, 1:500), GLAST (Frontier Institute, Af660, rabbit, 1:200), BLBP (Abcam, ab32423, rabbit, 1:500), FoxJ1 (eBioscience, 14-9965, mouse, 1:500), Galactocerebroside (GalC) (Millipore, MAB342, mouse, 1:500), RFP (MBL, PM005, rabbit, 1:1,000) and GFP (MBL, 598, rabbit, 1:2,000; Abcam, ab13970, chicken, 1:2,000). EdU labelling and staining were performed with the use of a Click-iT EdU Imaging Kit (Life Technologies). Immunoreactive cells were visualized by staining with secondary antibodies conjugated with Alexa Fluor 488, 568, 594, 633 or 647 (1:400, Life Technologies). A Zenon Alexa Fluor 488 Rabbit IgG Labeling Kit (Life Technologies) was used for immunostaining with two rabbit primary antibodies. Cell nuclei were stained with Hoechst 33342 (Sigma-Aldrich).

### Plasmid constructs and retrovirus production

The retroviral vectors pMX-IRES-EGFP (pMXIG)^61^ and pcUXIE were kindly provided by T. Kitamura and H. Song, respectively, and the plasmid pCAG-IRES-EGFP (pCAGIG) was provided by C. L. Cepko and T. Matsuda. Mouse Zbtb20 complementary DNA was obtained from OriGene. Mouse NFIA, human Brn2, mouse Zbtb16 and mouse Zbtb45 cDNAs were obtained from TransOMIC Technologies. Mouse Sox9 and rat Neurog2 cDNAs were amplified by PCR from cDNA libraries prepared from mouse NPCs or rat brain. The STAT3-C construct^62^ was kindly provided by J. F. Bromberg. An activated mutant of Notch1 (NICD) was described previously^14^. These various cDNAs were subcloned into pMXIG, pcUXIE, pCAGIG or pCAG. The pSIREN-shLuc (control shRNA), pSIREN-shZbtb20 (Zbtb20 shRNA), pSIREN-shSox9 (Sox9 shRNA) and pSIREN-shNFIA (NFIA shRNA) retroviral constructs were generated as specified by the manufacturer (BD Biosciences and Clontech). The pSIREN shRNA vector was modified to express GFP under the control of the phosphoglycerate kinase (*PGK*) gene promoter by replacement of the puromycin resistance gene with the *GFP* gene, thus allowing the simultaneous expression of GFP and the shRNA of interest. The target sequences were as follows: Zbtb20 shRNAs #1 and #2, 5′-GCTACAGCGACATCGAAATCC-3′ and 5′-GCCCAAAGGTGAAAGCTTTGA-3′, respectively; Sox9 shRNAs #1 and #2, 5′-GGAACAGACTCACATCTCTCC-3′ and 5′-GCTGTGGCTGGAGAGTATAAG-3′, respectively; and NFIA shRNAs #1 and #2, 5′-GCCCGAAAGCGGAAATACTTC-3′ and 5′-GCCAAGTTACGGAAAGATATC-3′, respectively. The shRNA-resistant mutant of Zbtb20 (mut-Zbtb20) was generated by introducing silent mutations (underlined) at the target sequence for shRNA #1 (5′-GCTACTCAGATATCGAGATTC-3′) with the use of a PrimeSTAR Mutagenesis Basal Kit (Takara). All viral vectors were introduced into the packaging cell lines Plat-E^61^ or Plat-GP by transfection with polyethyleneimine (Polysciences).

### Neurosphere culture and retrovirus infection

E11.5 or E16.5 mouse embryos were collected from timed pregnant mice and placed in an artificial cerebrospinal fluid (124 mM NaCl, 5 mM KCl, 1.3 mM MgCl_2_, 2 mM CaCl_2_, 26 mM NaHCO_3_ and 10 mM D-glucose). The forebrain or the spinal cord was removed from the rest of embryos under dissection microscope (Zeiss SV-11). The tissue was dissociated by incubation in a low-Ca^2+^, high-Mg^2+^ artificial cerebrospinal fluid (124 mM NaCl, 5 mM KCl, 3.2 mM MgCl_2_, 0.1 mM CaCl_2_, 26 mM NaHCO_3_, 10 mM D-glucose, 100 units per ml penicillin and 100 μg ml^−1^ streptomycin (Life Technologies)) containing 0.05% (w/v) trypsin (Sigma-Aldrich), 0.67 mg ml^−1^ hyaluronidase (Sigma-Aldrich) and 0.1 mg ml^−1^ deoxyribonuclease I (Roche) at 37 °C for 10 min. Subsequently, trypsin was neutralized with 0.7 mg ml^−1^ ovamucoid (Sigma-Aldrich) and the resultant tissue suspension was triturated mechanically to yield a single-cell suspension. The cells were filtered through a sterile nylon mesh (40 μm; Corning), washed twice with DMEM/F-12 (1:1) (Life Technologies) and were cultured in DMEM/F-12 supplemented with the B-27 supplement (Life Technologies), FGF2 (20 ng ml^−1^; Life Technologies), EGF (20 ng ml^−1^; Millipore) and heparin (2 μg ml^−1^; Sigma-Aldrich). To infect cells with virus, primary neurospheres were dissociated at day 2 after seeding and replated to yield secondary spheres. The cells were harvested at day 4 and subjected to retrovirus infection by incubation with high-titre virus, to yield an infection efficiency of 60–80%. For differentiation assay, the virus-infected neurospheres were dissociated, seeded in glass chambers coated with poly-D-lysine (100 μg ml^−1^; Sigma-Aldrich), cultured for 6 days without FGF2 and EGF, and subjected to immunostaining. For clonal analysis, the cells were plated at a clonal density (160 cells per mm^2^) and cultured with FGF2 for 3 days and without FGF2 for 4 days. For assay of proliferation, dividing cells were labelled with EdU (4 μM; Life Technologies) for 2 h. In some experiments, cells were treated with CNTF (50 ng ml^−1^; Sigma-Aldrich) or BMP4 (10 ng ml^−1^; R&D Systems).

### Cell culture and transfection

293T cells (ATCC) were maintained in DMEM (Wako) containing 10% fetal bovine serum (Thermo Scientific). The cells were transfected using polyethyleneimine. The medium was replaced 6 h after transfection and the cells were harvested at 48 h post transfection.

### Immunostaining

Cryosections (thickness of 16 μm) were prepared from mouse embryonic or postnatal forebrain and spinal cord or from adult (8–10 weeks of age) brain and spinal cord. Sections were incubated in a blocking solution (4% donkey serum, Millipore) at room temperature (RT) for 2 h, and incubated with primary antibodies overnight at RT and then with secondary antibodies at RT for 2 h. For *in utero* electroporation experiments, NPC differentiation in the cortex of P7 mouse brain was examined by quantifying the percentage of marker^+^ cells among GFP^+^ cells in defined areas (1.2 × 1.2 mm). Three to five coronal sections were selected from each animal, to represent comparable regions of the anterior and posterior parts of the forebrain. For immunocytochemistry, cultured cells were fixed with 4% paraformaldehyde for 20 min and incubated in a blocking solution (10% fetal bovine serum, Thermo Scientific) for 30 min at RT. The cells were incubated with primary antibodies for 3 h and then with secondary antibodies for 1 h at RT.

### *In utero* and postnatal electroporation

For *in utero* electroporation, pCAGIG-Zbtb20 or pCAG-Zbtb20 with pCAG-mCherry vectors (2.5 mg ml^−1^) were injected into the lateral ventricle of the mouse brain at E15.5. Electrodes were positioned at the flanking ventricular regions of each embryo and four pulses of 40 V for 50 ms were applied at intervals of 950 ms with an electroporator (CUY21E, Tokiwa Science). For postnatal electroporation, pSIREN knockdown vectors for Zbtb20 and pCAGIG-mut-Zbtb20 (each at 2.5 mg ml^−1^) or pSIRENΔGFP-shZbtb20 (without *GFP* gene) with pCAG-mCherry were injected into the lateral ventricle of P0 mouse pups. Eight pulses of 80 V for 50 ms were applied at intervals of 950 ms. The brain was isolated at E18.5, P3 or P7.

### RT and real-time PCR analysis

Total RNA was isolated with the use of the TRIzol reagent (Life Technologies) or an RNeasy Mini Kit (Qiagen) and portions (1 μg) of the RNA were subjected to RT with the use of a ReverTra Ace qPCR RT Kit (TOYOBO). The resulting cDNA was subjected to real-time PCR analysis in a Roche LightCycler with SYBR Premix Ex Taq (Takara) or in an Applied Biosystems 7500 Real Time PCR System with Power SYBR Green PCR Master Mix (Life Technologies). Glyceraldehyde-3-phosphate dehydrogenase mRNA was examined as an internal control. The primers (sense and antisense, respectively) were as follows: mouse Zbtb20, 5′-AACGCAATGAATCCGAGGAGT-3′ and 5′-CCCAAACTGTTGCTCCACTGA-3; mouse Sox9, 5′-CCAACATTGAGACCTTCGACGT-3′ and 5′-ATGCCGTAACTGCCAGTGTAGG-3′; mouse NFIA, 5′-TTGGACCTCGTCATGGTGATC-3′ and 5′-TGGACACAGAGCCCTGGATTA-3′; mouse Brn2, 5′-GCAAGCTGAAGCCTTTGTTGA-3′ and 5′-GGTCCGCTTTTTCCGTTTG-3′; mouse Brn4, 5′-GTTGGAACAGTTCGCCAAACA-3′ and 5′-TGCGAGAACACGTTGCCAT-3′; mouse Sox4, 5′-GGACAGCGACAAGATTCCGTT-3′ and 5′-TGCCCGACTTCACCTTCTTTC-3′; mouse Sox11, 5′-ATGGTGTGGTCCAAGATCGAG-3′ and 5′-TCAGCATCTTCCAGCGCTT-3′; mouse NFIB, 5′-AGAAGCCCGAAATCAAGCAGA-3′ and 5′-GCCAGTCACGGTAAGCACAAA-3′; mouse Brn1, 5′-CTGGAGCAGTTCGCTAAGCAGT-3′ and 5′-TGCGAGAACACGTTGCCATA-3′; mouse Zhx2, 5′-GGAGCACATCCGAATCTGGTT-3′ and 5′-TGCCGTTAAACATCTTCTTCCG-3′; mouse Sox10, 5′-TCTCACGACCCCAGTTTGACT-3′ and 5′-GCCCCATGTAAGAAAAGGCTG-3′; mouse Zbtb16, 5′-TGCCCAGTTCTCAAAGGAGGA-3′ and 5′-GCTTTGTGCCTGAAAGCGTTT-3′; mouse Zbtb45, 5′-AGCTCGCGGAAAAACTACACC-3′ and 5′-CAGGTAATCGCGTAGCGAGAA-3′; and mouse glyceraldehyde-3-phosphate dehydrogenase, 5′-GCAAAGTGGAGATTGTTGCCAT-3′ and 5′-CCTTGACTGTGCCGTTGAATTT-3′.

### Luciferase assay

The *Gfap* promoter–Luc (GF1L-pGL3) plasmid^15^ was kindly provided by K. Nakashima. The mouse *Brn2* promoter^63^ from positions −2355 to +60 was cloned from mouse NPCs and inserted into the pGL3 vector (Promega). NPCs were transfected with *Gfap* promoter–Luc or *Brn2* promoter–Luc plasmids; with expression plasmids for Zbtb20, Sox9, NFIA, Neurog2 or STAT3-C; with knockdown plasmids for Sox9 or NFIA; and with the pRL-SV40 plasmid encoding *Renilla* luciferase (Promega) with the use of Lipofectamine 2000 (Life Technologies). The cells were cultured for 48 h with FGF2, after which cell extracts were prepared and assayed for luciferase activity with the use of a Dual-Luciferase Reporter Assay System (Promega). Firefly luciferase activity was normalized by that of *Renilla* luciferase.

### ChIP assay

NPCs were infected with control or TY1-tagged Zbtb20 viruses and the ChIP assay was performed 48 h after infection with the use of a ChIP-IT Express Enzymatic Kit (Active Motif) with some modifications. The cells were fixed with 1% formaldehyde and the cross-linking reaction was terminated by the addition of glycine. The cross-linked chromatin was sheared into fragments by enzymatic digestion at 37 °C for 10 min and the fragments were then incubated overnight at 4 °C with antibodies to TY1 (Diagenode, MAb-054-050, 2 μg) or with normal mouse IgG (Santa Cruz Biotechnology, sc2025, 2 μg). After the addition of Dynabeads Protein G (Life Technologies), the mixture was incubated with rotation for 1 h. The beads were then isolated and washed consecutively with a low-salt solution (0.1% SDS, 1% Triton X-100, 2 mM EDTA, 20 mM Tris-HCl pH 8.1 and 150 mM NaCl), a high-salt solution (0.1% SDS, 1% Triton X-100, 2 mM EDTA, 20 mM Tris-HCl pH 8.1 and 500 mM NaCl) and a LiCl solution (0.25 M LiCl, 1% Nonidet P-40, 1% sodium deoxycholate, 1 mM EDTA and 10 mM Tris-HCl pH 8.1) and then twice with a Tris-EDTA solution (10 mM Tris-HCl pH 8.0 and 1 mM EDTA). Immune complexes were eluted from the beads with a solution containing 10 mM dithiothreitol, 1% SDS and 0.1 M NaHCO_3_, after which NaCl was added to a final concentration of 0.2 M and the eluate was incubated at 65 °C overnight. Proteins were eliminated by digestion with proteinase K (Life Technologies) at 45 °C for 1 h and the remaining DNA was purified with the use of a QIAquick spin column (Qiagen). The eluted DNA was subjected to real-time PCR analysis in an Applied Biosystems 7500 Real Time PCR System with Power SYBR Green PCR Master Mix (Life Technologies). The amount of target genomic DNA was normalized by that of the input. The PCR primers (sense and antisense, respectively) were as follows: *Brn2* gene R1, 5′-GCGCAGACACTTAGCGTCCT-3′ and 5′-AAGAACTCCCTCCCCCAGG-3′; R2, 5′-GCTGTCCCCTCCATACACTTTC-3′ and 5′-CTGCGAATCTCCACGTGTTCT-3′; R3, 5′-GGAGGCCATTTGTGATCCC-3′ and 5′-TCAAAAGTTATTGCGGGTAAAGGA-3′; R4, 5′-GATCTTCCTCCCTTTTGCCC-3′ and 5′-AGGCCTGTCTGTGCAACTTTC-3′; R5, 5′-GGGAGATATTAGTCTAGCTTTGGCTC-3′ and 5′-CCAACGTCATTTAAACCTAGAACGA-3′; R6, 5′-CTGGGAGGTTGCTAGCGGTA-3′ and 5′-CATTGGCTCTGCGCCCT-3'; and R7, 5′-CACCCTGTACGGCAACGTG-3′ and 5′-GCCTCAAACCTGCAGATGGT-3′. *Sox10* gene region −6309 to −6259, 5′-CTTGCTCAGGTGCTGAGGTG-3′ and 5′-GATGACAGATTGCCGAGGGT-3′; −2183 to −2133, 5′-AAAAAACTGGTCCCTATGGCC-3′ and 5′-GGCAATCGGGATACCTTCTG-3′; −523 to −473, 5′-TTATCCAGCTGCGGTCCTG-3′ and 5′-TCTCCAACACAGCTGACCCA-3′; −231 to −181, 5′-AATATCCCCATGCTCACACCA-3′ and 5′-TCTCCCTGGACTCAGCTTGG-3′; −11 to +43, 5′-TGCAGCGGCTCAGTCAGTC-3′ and 5′-ATCCTCACCACCAAACACCC-3′; and +320 to +370, 5′-TCTGCTCCCCTAGGCTGTCA-3′ and 5′-TTCGCTCACCTCTCTTTCCC-3′.

### Immunoprecipitation and western blot analysis

The transfected cells were lysed in a lysis buffer (50 mM HEPES–NaOH pH 7.5, 50 mM NaCl, 0.5% Triton X-100, 1 mM EDTA, 1 mM EGTA, 1 mM dithiothreitol, 1 mM phenylmethylsulfonyl fluoride, 0.5% protease inhibitor cocktail (Sigma-Aldrich), 1 mM Na_3_VO_4_, 10 mM NaF and 20 mM β-glycerophosphate) and the cell lysates were centrifuged at 15,000 r.p.m. for 10 min at 4 °C. The supernatants were incubated for 3 h at 4 °C with anti-FLAG M2 affinity gel (Sigma-Aldrich). The immunoprecipitates were washed four times with a lysis buffer and subjected to immunoblotting with antibodies for Sox9 (Millipore, AB5535, rabbit, 1:1,000), NFIA (Abcam, ab41851, rabbit, 1:1,000) and FLAG (Sigma-Aldrich, F1804, mouse, 1:1,000). Immunoreactive bands were visualized with secondary horseradish peroxidase-conjugated antibodies (Promega, W4011, rabbit, 1:4,000; Promega, W4021, mouse, 1:4,000) and ECL reagents (GE Healthcare).

### Microarray analysis

NPCs derived from E11.5 mouse forebrain were infected with control or Zbtb20 viruses and virus-infected NPCs were isolated 3 days after infection. Sample preparation and microarray analysis with an Affymetrix Gene-Chip Mouse Gene 1.0 ST Array were performed by Cell Innovator. The microarray data are available in the Gene Expression Omnibus database (accession number GSE66583).

### Statistical analysis

Quantitative data are presented as means±s.d. and were analysed with the two-tailed unpaired Student's *t*-test. A *P*-value of <0.05 was considered statistically significant.

## Additional information

**How to cite this article**: Nagao, M. *et al*. Zbtb20 promotes astrocytogenesis during neocortical development. *Nat. Commun.* 7:11102 doi: 10.1038/ncomms11102 (2016).

## Supplementary Material

Supplementary Information Supplementary Figures 1-16
