# Supplementary material for: Zbtb20 promotes astrocytogenesis during neocortical development
Source: Nat Commun. 2016 Mar 22;7:11102. doi: 10.1038/ncomms11102 (PMC4804180; doi:10.1038/ncomms11102)
Supplement: Supplementary Information — Supplementary Figures 1-16 [file ncomms11102-s1.pdf]

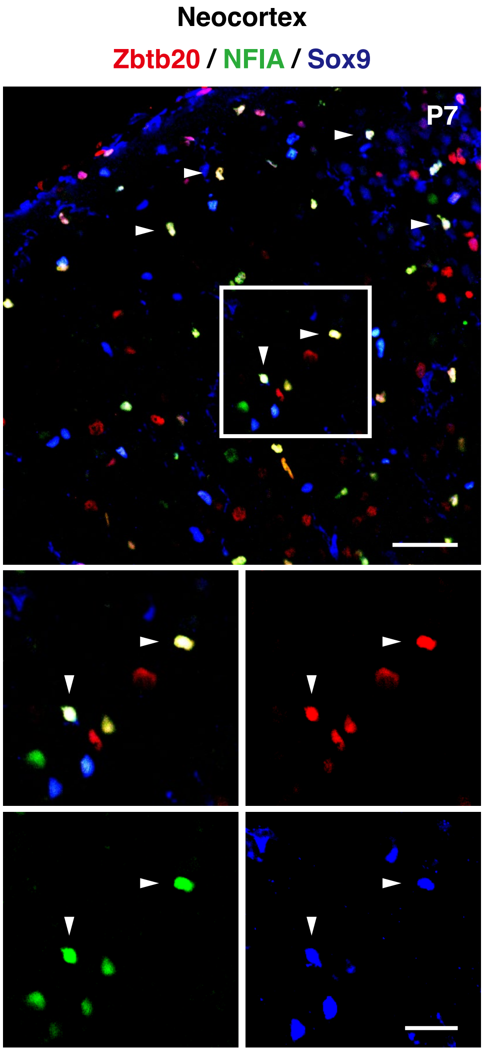

Supplementary Figure 1. Expression of Zbtb20, NFIA, and Sox9 in the mouse neocortex at P7. The lower panels are higher magnification views of the boxed area. Arrowheads indicate triple-positive cells. Scale bars, 50 and 25 μm in the main panel and higher magnification images, respectively.

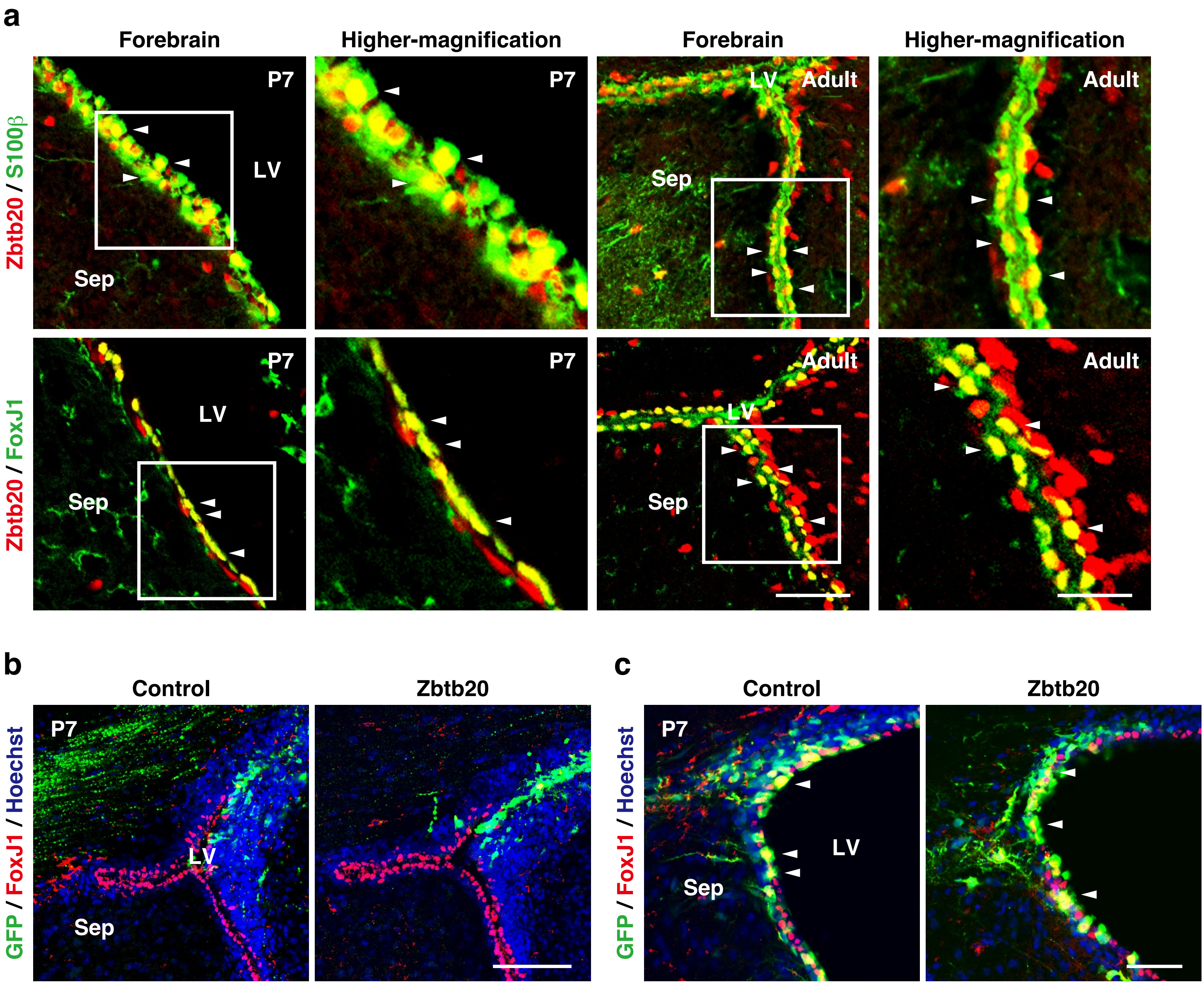

Supplementary Figure 2. Expression of Zbtb20 in ependymal cells. (**a**) Coexpression of Zbtb20 and either S100β or FoxJ1 in the P7 and adult forebrain. Right panels of each pair are higher magnification views of the boxed areas. (**b, c**) Expression plasmids for GFP alone (control) or for both GFP and Zbtb20 were injected into the lateral ventricle of the E15.5 mouse forebrain in utero and were introduced into the dorsolateral region of the neocortex (**b**) and the medial dorsoventral boundary of brains (**c**) by electroporation. The brain was isolated at P7 and subjected to immunostaining for FoxJ1 and GFP. LV, lateral ventricle; Sep, septum. Arrowheads indicate double-positive cells (**a, c**). Scale bars, 50 μm (**a, c**), 100 μm (**b**), and 25 μm (higher magnification images in **a**).

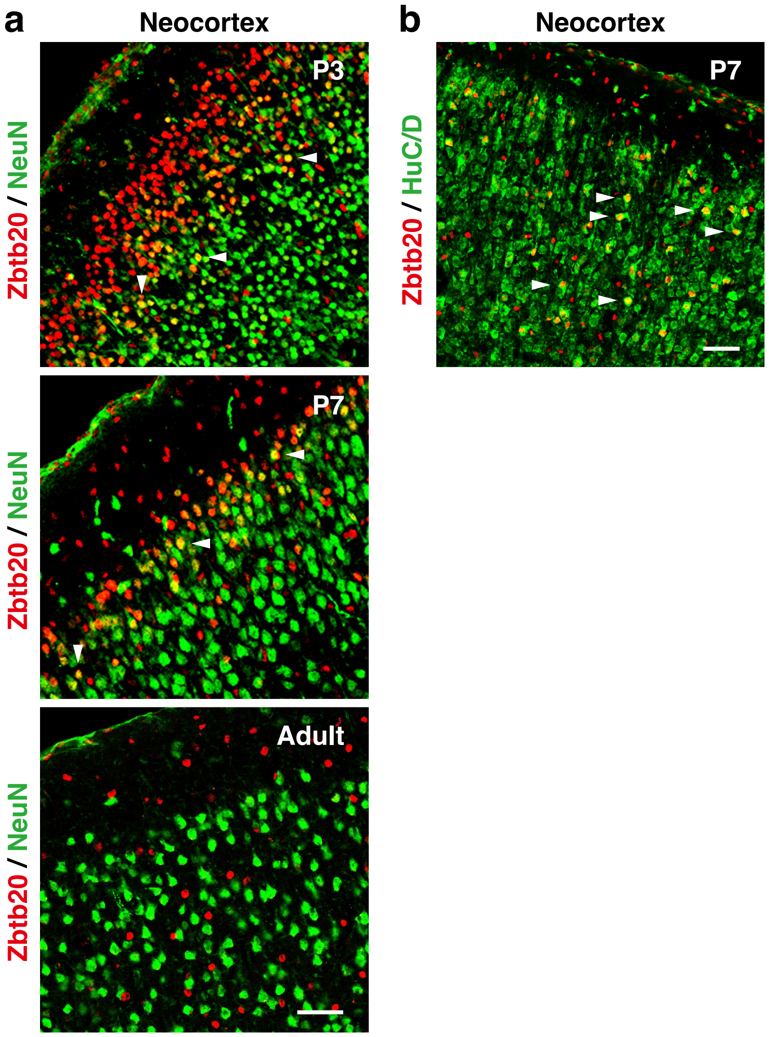

Supplementary Figure 3. Transient expression of Zbtb20 in NeuN+ neurons and HuC/D+ immature neurons. (**a**) Coexpression of Zbtb20 and NeuN in the P3, P7, and adult neocortex. (**b**) Coexpression of Zbtb20 and HuC/D in the P7 neocortex. Arrowheads indicate double-positive cells. Scale bars, 50 μm.

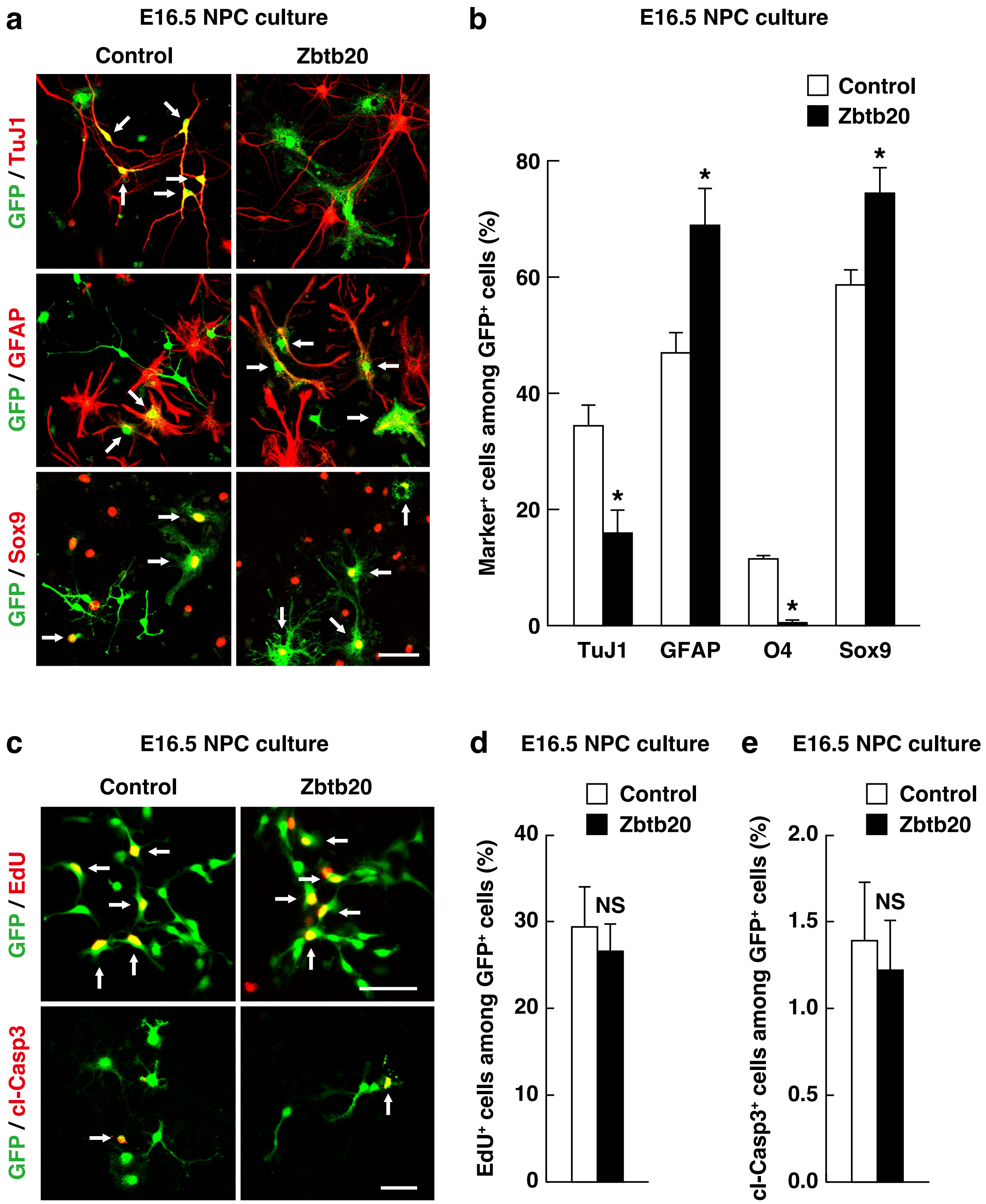

Supplementary Figure 4. Effects of Zbtb20 on the proliferation, survival, and differentiation of NPCs in vitro. (**a**, **b**) NPCs derived from E16.5 mouse forebrain were infected with retroviruses encoding GFP alone (control) or both GFP and Zbtb20. Two days after infection, the cells were induced to differentiate for 6 days and then stained for TuJ1, GFAP, O4, Sox9, and GFP (**a**). Arrows indicate marker$^+$/GFP$^+$ cells. The percentages of marker$^+$ cells among total GFP$^+$ cells were determined as means ± s.d. (n = 3) (**b**). (**c**–**e**) E16.5 NPCs infected with control or Zbtb20 retroviruses were labeled with EdU for 2 h in the presence of FGF2 and EGF. The cells were stained for EdU and cleaved caspase 3 (cl-Casp3) 1 day after plating (**c**). Arrows indicate EdU$^+$/GFP$^+$ cells or cleaved caspase 3$^+$/GFP$^+$ cells. The percentages of EdU$^+$ (**d**) or cleaved caspase 3$^+$ (**e**) cells among GFP$^+$ cells were determined as means ± s.d. (n = 3). *$P < 0.01$ versus the corresponding control value; NS, non-significant; $P = 0.45$ (**d**) and 0.53 (**e**) versus corresponding control value. Scale bars, 50 μm.

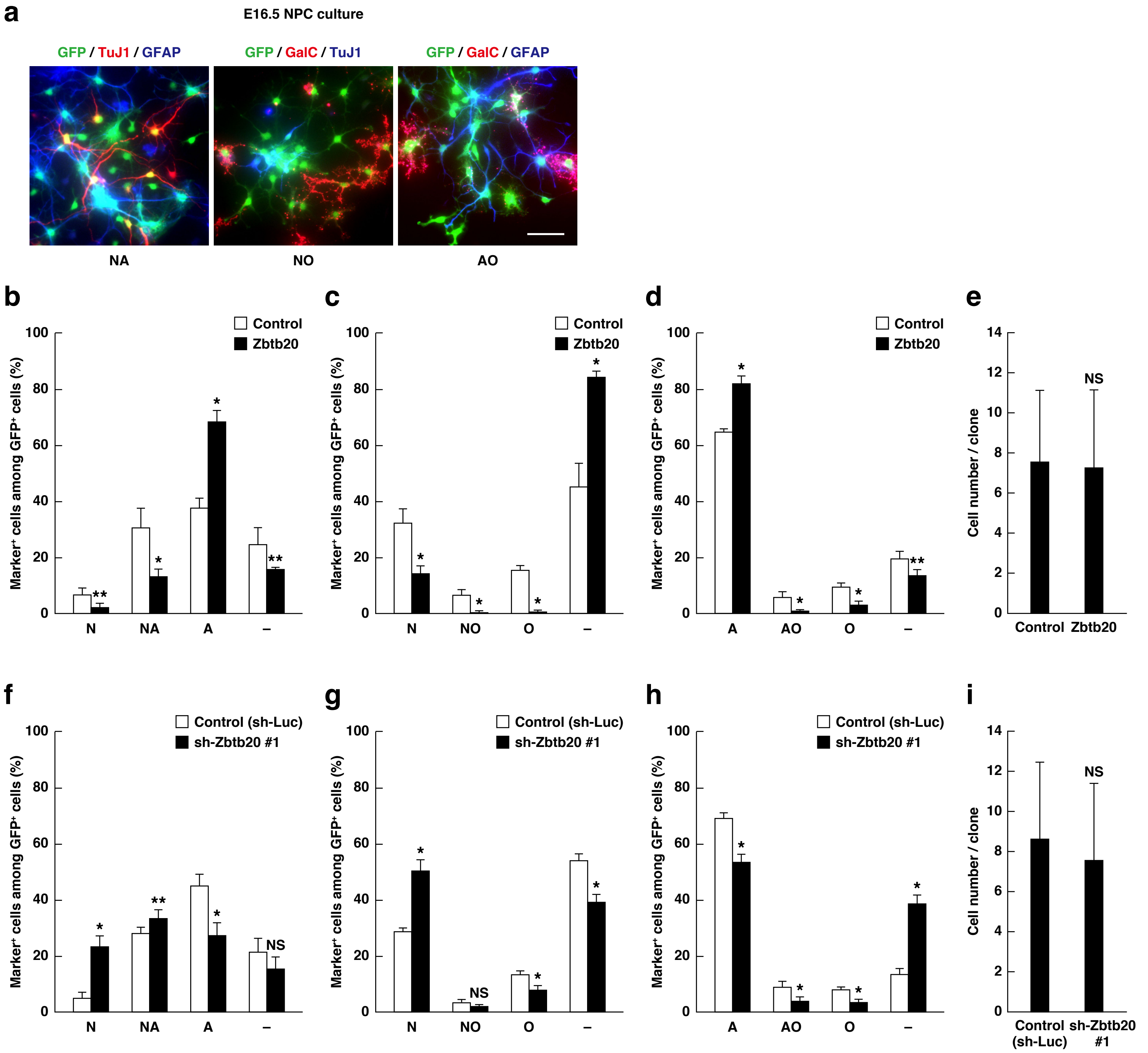

Supplementary Figure 5. Clonal analysis of Zbtb20-overexpressing and Zbtb20 knockdown cells. (**a–i**) The control, Zbtb20-overexpressing or Zbtb20 knockdown cells were plated at a clonal density and cultured with FGF2 for 3 days and without FGF2 for 4 days. The percentages of neuron clones (N) which contain TuJ1$^+$ cells and do not contain GFAP$^+$ cells, neuron and astrocyte clones (NA) which contain both TuJ1$^+$ cells and GFAP$^+$ cells, or astrocyte clones (A) which contain GFAP$^+$ cells and do not contain TuJ1$^+$ cells among total clones were quantified (**b, f**). The percentages of neuron clones (N) which contain TuJ1$^+$ cells and do not contain GalC$^+$ cells, neuron and oligodendrocyte clones (NO) which contain both TuJ1$^+$ cells and GalC$^+$ cells, or oligodendrocyte clones (O) which contain GalC$^+$ cells and do not contain TuJ1$^+$ cells among total clones were quantified (**c, g**). The percentages of astrocyte clones (A) which contain GFAP$^+$ cells and do not contain GalC$^+$ cells, astrocyte and oligodendrocyte clones (AO) which contain both GFAP$^+$ cells and GalC$^+$ cells, or oligodendrocyte clones (O) which contain GalC$^+$ cells and do not contain GFAP$^+$ cells among total clones were quantified (**d, h**). The cell number per clone was also determined (**e, i**). Data are means ± s.d. (n = 3). *$P < 0.01$, **$P < 0.05$ versus corresponding control value; NS, non-significant; $P = 0.92$ (**e**), 0.19 (**f**), 0.15 (**g**) and 0.75 (**i**) versus corresponding control value. Scale bar, 50 μm.

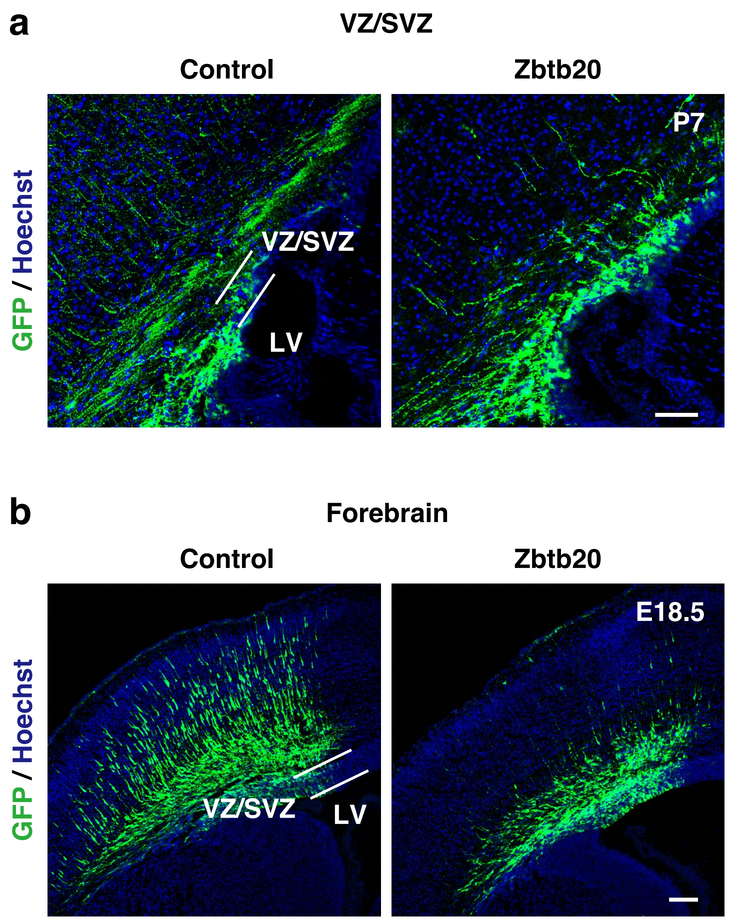

Supplementary Figure 6. The distribution pattern of Zbtb20-overexpressing cells around the VZ/SVZ and in the neocortex. (**a**, **b**) Expression plasmids for GFP alone (control) or for both GFP and Zbtb20 were injected into the lateral ventricle of E15.5 mouse forebrain in utero and were introduced into the dorsolateral region of the neocortex by electroporation. The brains were isolated at P7 (**a**) or E18.5 (**b**) and subjected to immunostaining for GFP. LV, lateral ventricle; SVZ, subventricular zone; VZ, ventricular zone. Scale bars, 75 μm (**a**) and 100 μm (**b**).

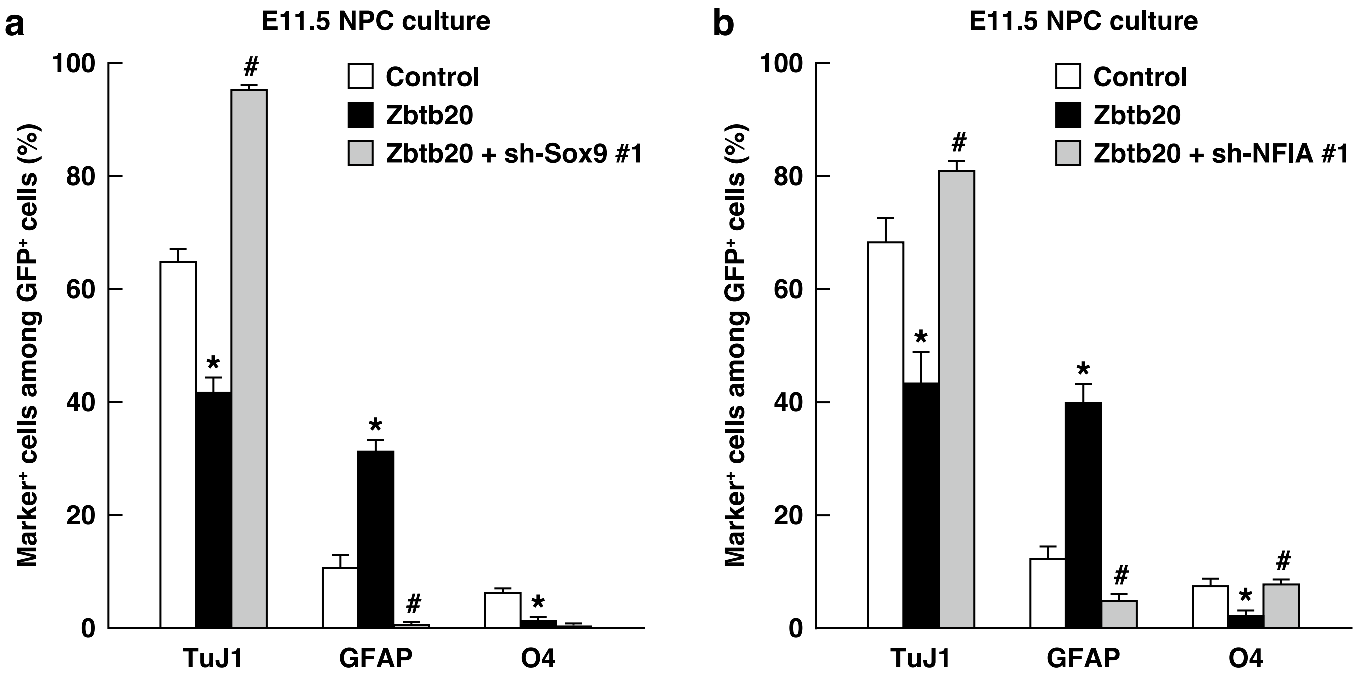

Supplementary Figure 7. Effects of knockdown of Sox9 or NFIA on the promotion of astrocyte differentiation by Zbtb20. (**a, b**) E11.5 NPCs were infected with control, Zbtb20, or Zbtb20 plus either sh-Sox9 #1 (**a**) or sh-NFIA #1 (**b**) retroviruses, cultured without FGF2 and EGF for 6 days, and subjected to immunostaining for TuJ1, GFAP, O4, and GFP. The percentages of marker$^+$ cells among total GFP$^+$ cells were determined as means ± s.d. (n = 3). *P < 0.01 versus corresponding control value; #P < 0.01 versus value for Zbtb20 alone.

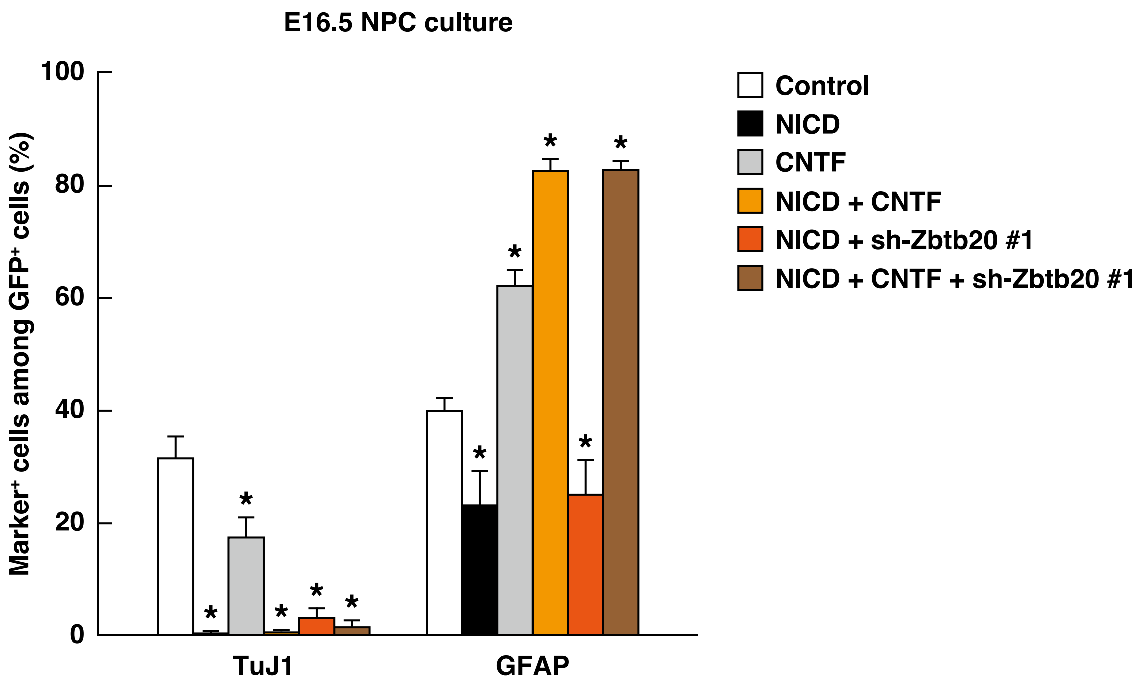

Supplementary Figure 8. Effect of Zbtb20 knockdown on astrocyte differentiation induced by NICD and CNTF. E16.5 NPCs were infected with control, NICD, or NICD plus sh-Zbtb20 #1 retroviruses, cultured without or with CNTF for 6 days, and then stained for TuJ1, GFAP, and GFP for determination of the percentages of marker$^+$ cells among total GFP$^+$ cells (means ± s.d., n = 3). *P < 0.01 versus corresponding control value.

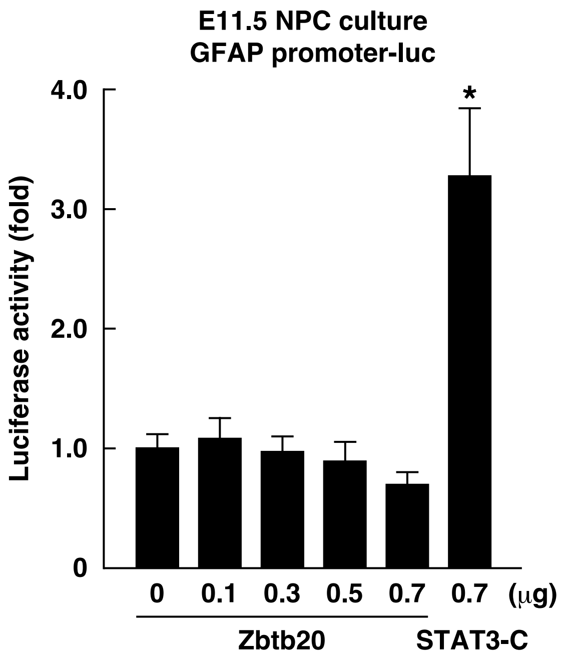

Supplementary Figure 9. Effect of Zbtb20 on the activity of the *Gfap* promoter. Luciferase reporter assay of relative *Gfap* promoter activity in NPCs transfected with the indicated amounts of expression plasmids for Zbtb20 or STAT3-C (positive control). Data are means ± s.d. (n = 3). *P < 0.01 versus corresponding control.

a

Astrocytic genes	Fold change (Zbtb20/Control)
Aldh1L1	0.95
Aldoc	0.85
CNTFR	1.05
FGFR3	1.00
GFAP	1.29
GLAST	0.94
GLT-1	1.00
gp130	0.87
NFIA	0.82
NFIX	0.98
S100β	1.55
Sox9	0.87
STAT1	0.97
STAT3	0.98

b

Downregulated neuronal genes	Fold change (Zbtb20/Control)
Brn2 (Pou3f2)	0.56
Brn4 (Pou3f4)	0.78
NFIB	0.72
Sox4	0.70
Sox11	0.64

Supplementary Figure 10. Microarray analysis of Zbtb20-overexpressing NPCs. (**a, b**) E11.5 NPCs were infected with control or Zbtb20 viruses. The virus-infected NPCs were isolated 3 days after infection and subjected to microarray analysis. Fold changes in the expression level of the indicated astrocytic (**a**) and neuronal (**b**) genes in Zbtb20-overexpressing NPCs relative to control are shown.

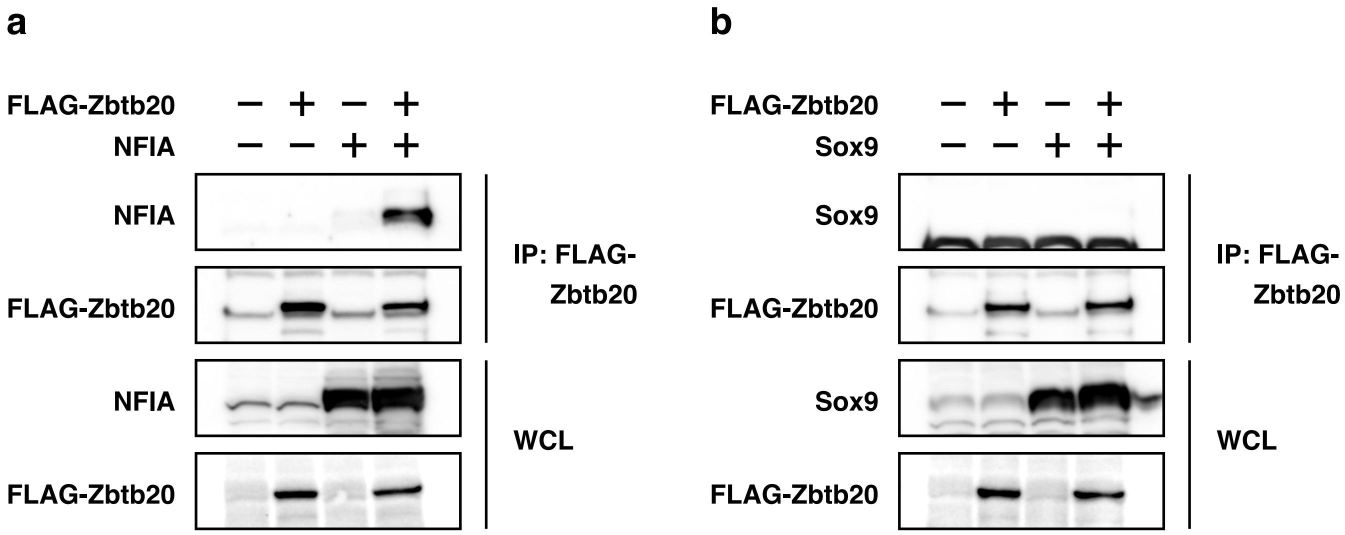

Supplementary Figure 11. Zbtb20 interacts with NFIA. (**a, b**) 293T cells were transfected with plasmids for FLAG-tagged Zbtb20, Sox9, or NFIA, as indicated. The cell lysates were subjected to co-immunoprecipitation and western blot analysis. IP, immunoprecipitation; WCL, whole cell lysate.

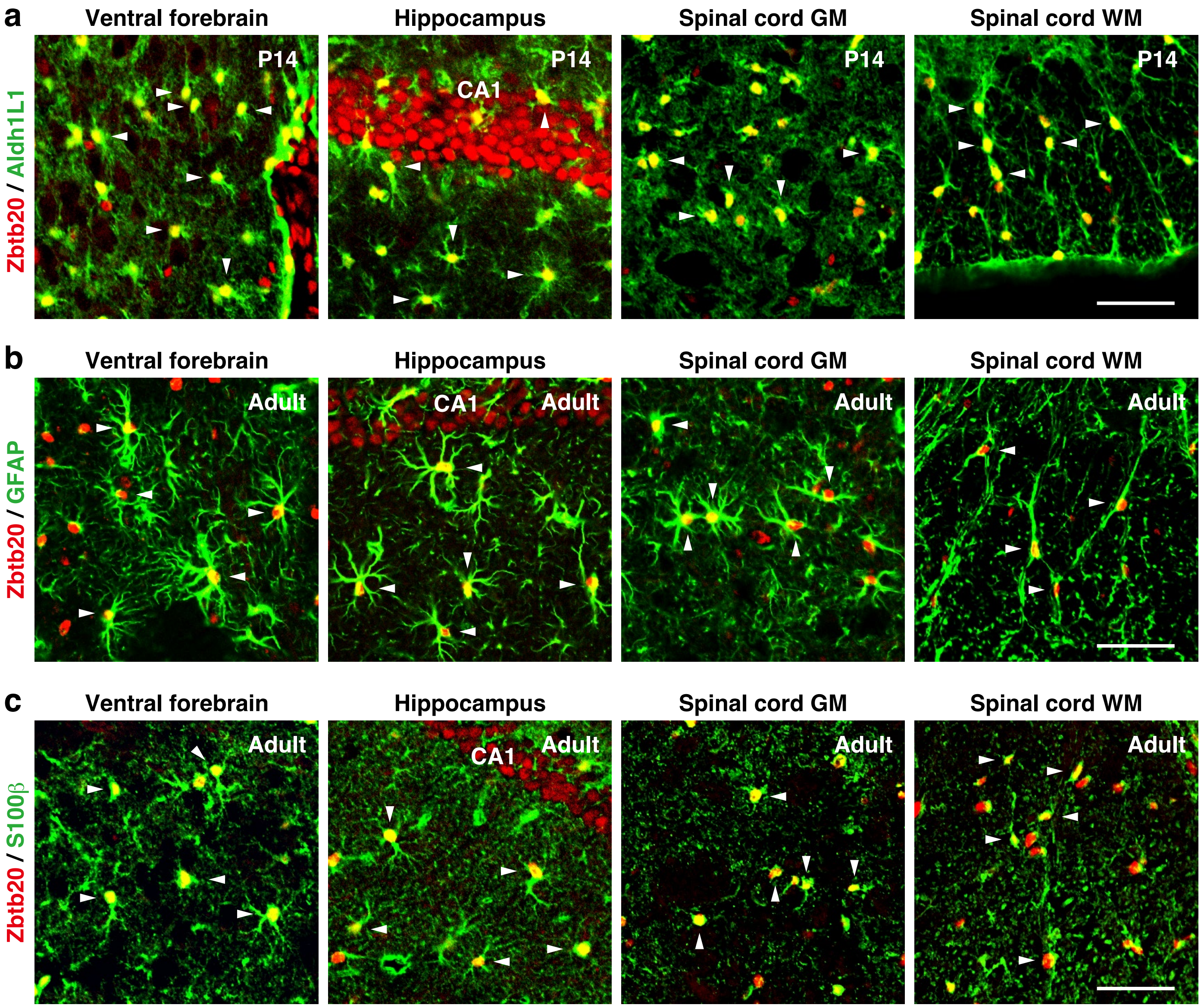

Supplementary Figure 12. Expression of Zbtb20 in the CNS regions other than neocortex. (**a**) Double staining for Zbtb20 and Aldh1L1-GFP in the P14 ventral forebrain, hippocampus, and spinal cord. (**b, c**) Double staining for Zbtb20 and either GFAP (**b**) or S100β (**c**) in the adult ventral forebrain, hippocampus, and spinal cord. CA1, hippocampal CA1 sector; GM, gray matter; WM, white matter. Arrowheads indicate double-positive cells. Scale bars, 50 μm.

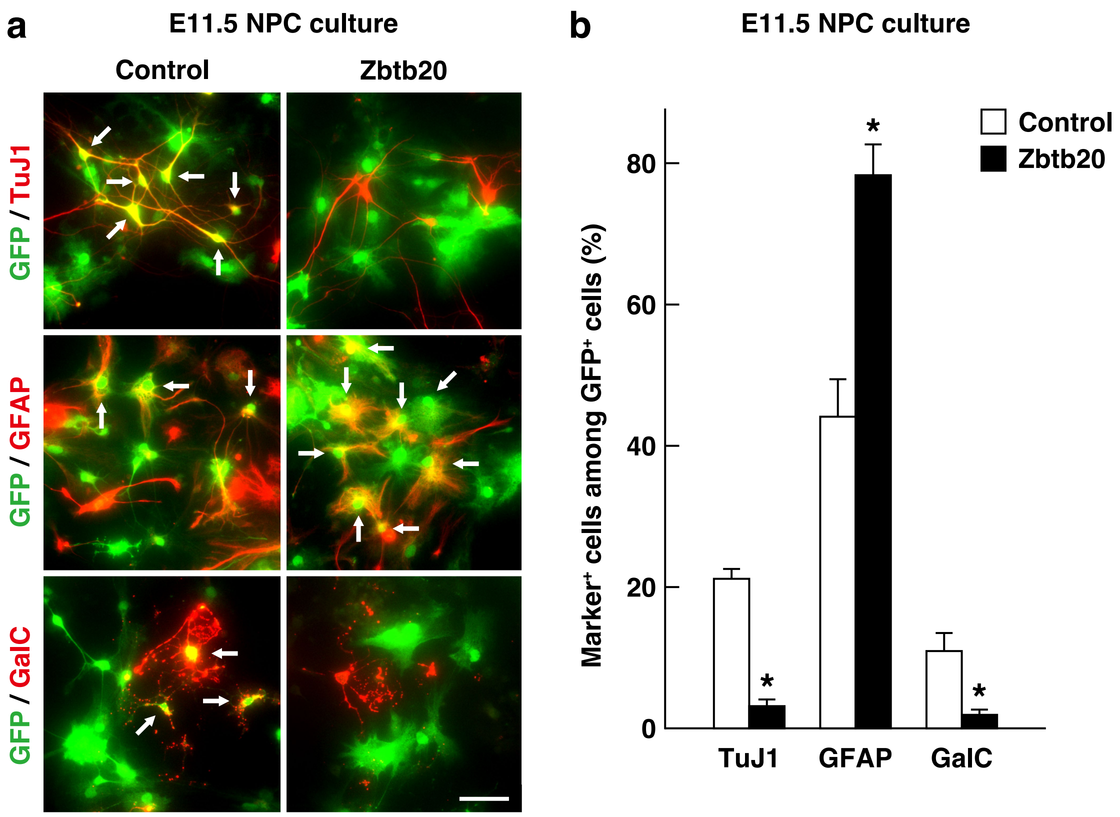

Supplementary Figure 13. Effects of Zbtb20 on the differentiation of spinal cord NPCs in vitro. (**a, b**) NPCs derived from E11.5 mouse spinal cord were infected with retroviruses encoding GFP alone (control) or both GFP and Zbtb20. Two days after infection, the cells were induced to differentiate for 6 days and then stained for TuJ1, GFAP, GalC, and GFP (**a**). Arrows indicate marker$^+$/GFP$^+$ cells. The percentages of marker$^+$ cells among total GFP$^+$ cells were determined as means ± s.d. ($n = 3$) (**b**). *$P < 0.01$ versus the corresponding control value. Scale bar, 50 μm.

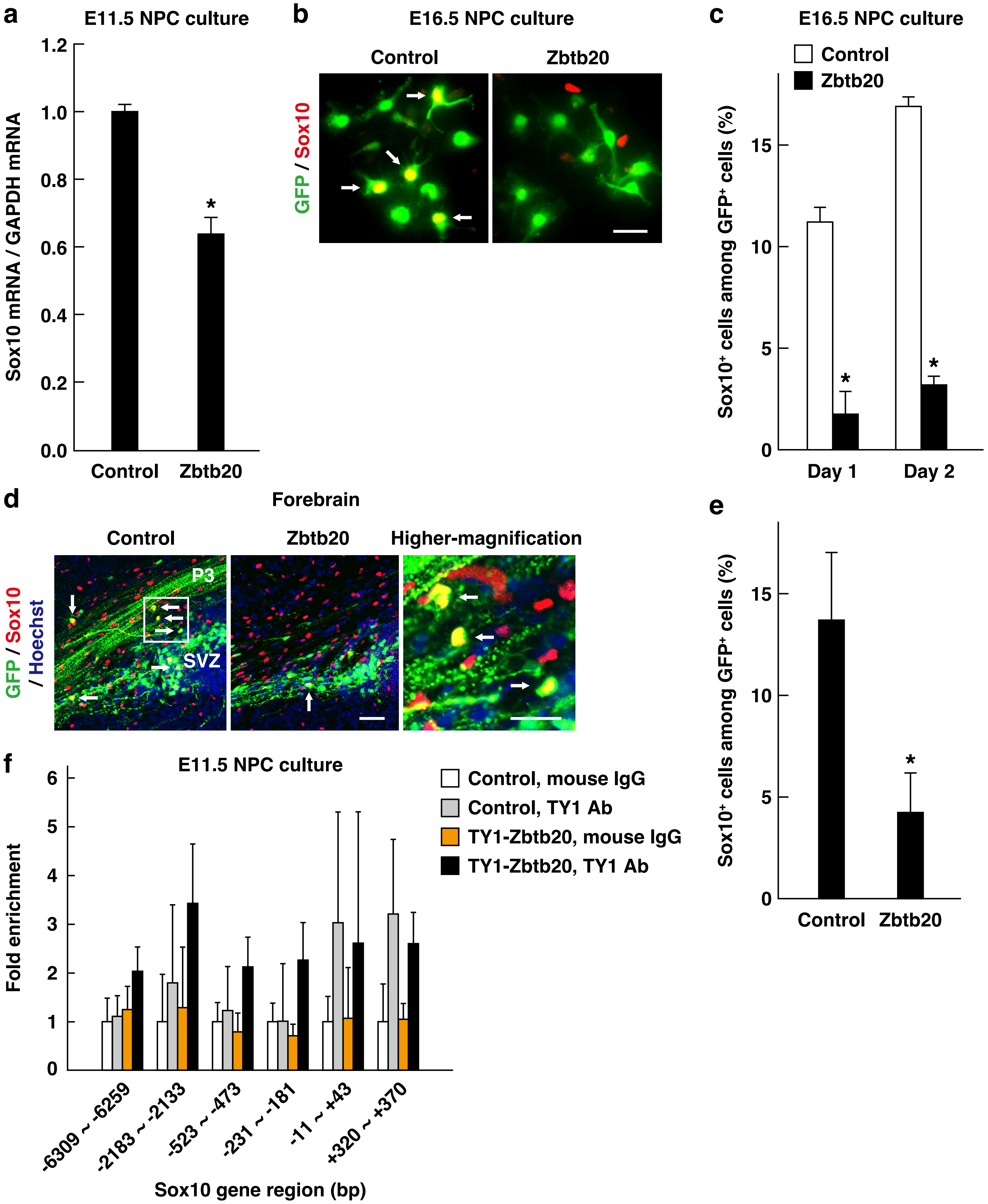

Supplementary Figure 14. Zbtb20 suppresses oligodendrocyte differentiation. (**a**) Quantitative RT-PCR analysis of relative Sox10 mRNA abundance in Zbtb20-overexpressing and control NPCs. Data are means ± s.d. (n = 3). (**b, c**) E16.5 NPCs were infected with retroviruses encoding GFP alone (control) or both GFP and Zbtb20. Two days after infection, the cells were induced to differentiate for 1 or 2 days and then stained for Sox10 and GFP (**b**). The percentages of $Sox10^+$ cells among total GFP^+ cells were determined as means ± s.d. (n = 3) (**c**). (**d, e**) Expression plasmids for GFP alone (control) or for both GFP and Zbtb20 were injected into the lateral ventricle of the E15.5 mouse forebrain in utero and were introduced into the lateral dorsoventral boundary of brains by electroporation. The brain was isolated at P3 and subjected to immunostaining for Sox10 and GFP (**d**). The percentages of $Sox10^+$ cells among total GFP^+ cells were determined as means ± s.d. (n = 10) (**e**). (**f**) ChIP analysis of Zbtb20 binding to the Sox10 gene region in NPCs. Six different regions of the Sox10 locus were tested in control cells and cells expressing TY1-tagged Zbtb20. Data are expressed as fold enrichment relative to the corresponding value for control cells and normal mouse immunoglobulin G (IgG). Data are means ± s.d. (n = 3). SVZ, subventricular zone; bp, base pair; Ab, antibody. The boxed region in d is shown at higher magnification in the right-most panel. Arrows indicate $Sox10^+/GFP^+$ cells (**b, d**). Scale bars, 50 μm (**d**) and 25 μm (**b** and higher magnification image in **d**). *$P < 0.01$ versus the corresponding control value.

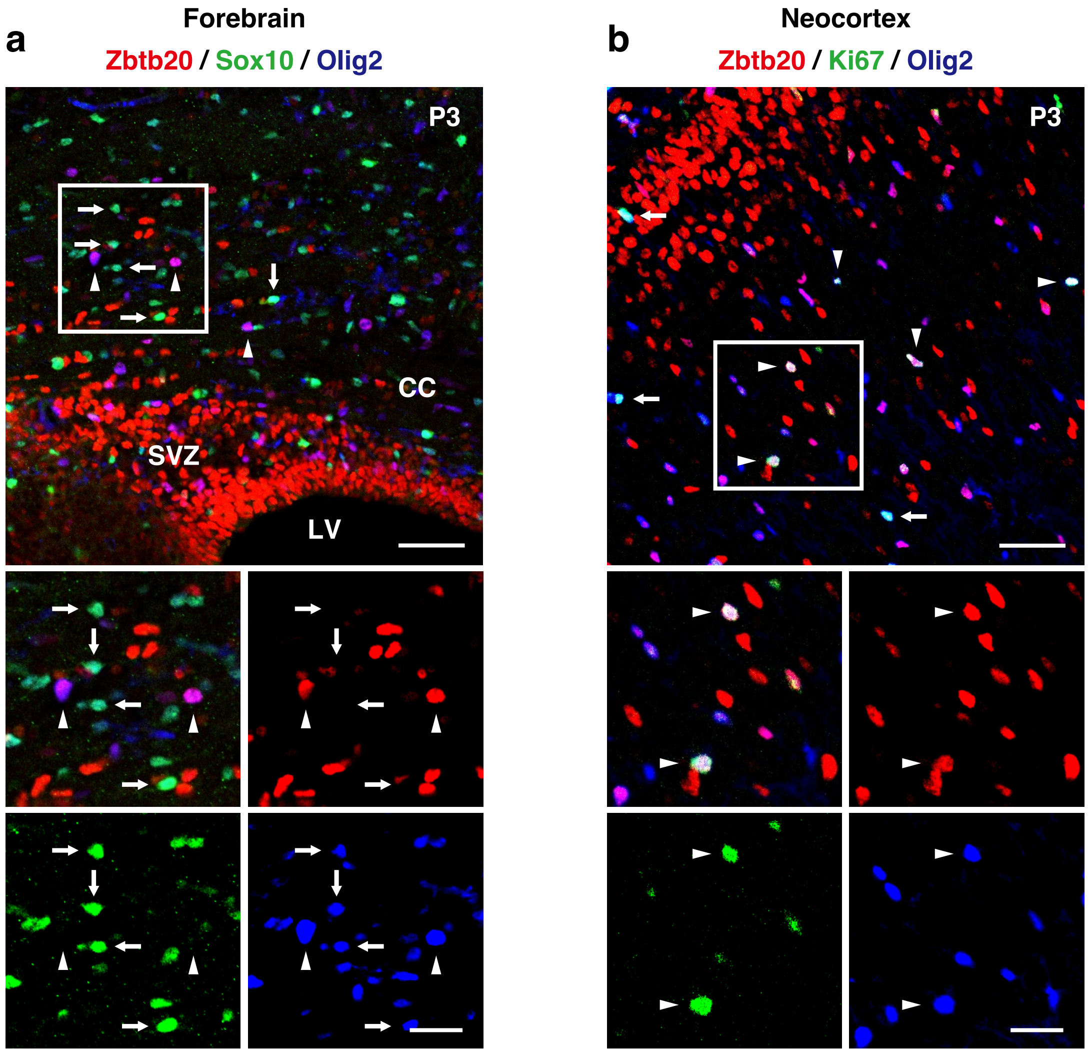

Supplementary Figure 15. Zbtb20⁺/Olig2⁺ cells are astrocyte precursors in the developing neocortex. (**a**) Zbtb20$^+$/Olig2$^+$/Sox10$^-$ cells (arrowheads) and Zbtb20$^-$/Olig2$^+$/Sox10$^+$ cells (arrows) in the P3 neocortex. (**b**) Zbtb20$^+$/Olig2$^+$/Ki67$^+$ cells (arrowheads) and Zbtb20$^-$/Olig2$^+$/Ki67$^+$ cells (arrows) in the P3 neocortex. CC, corpus callosum; LV, lateral ventricle; SVZ, subventricular zone. The lower panels are higher magnification views of the boxed areas. Scale bars, 50 μm or 25 μm (higher magnification images).

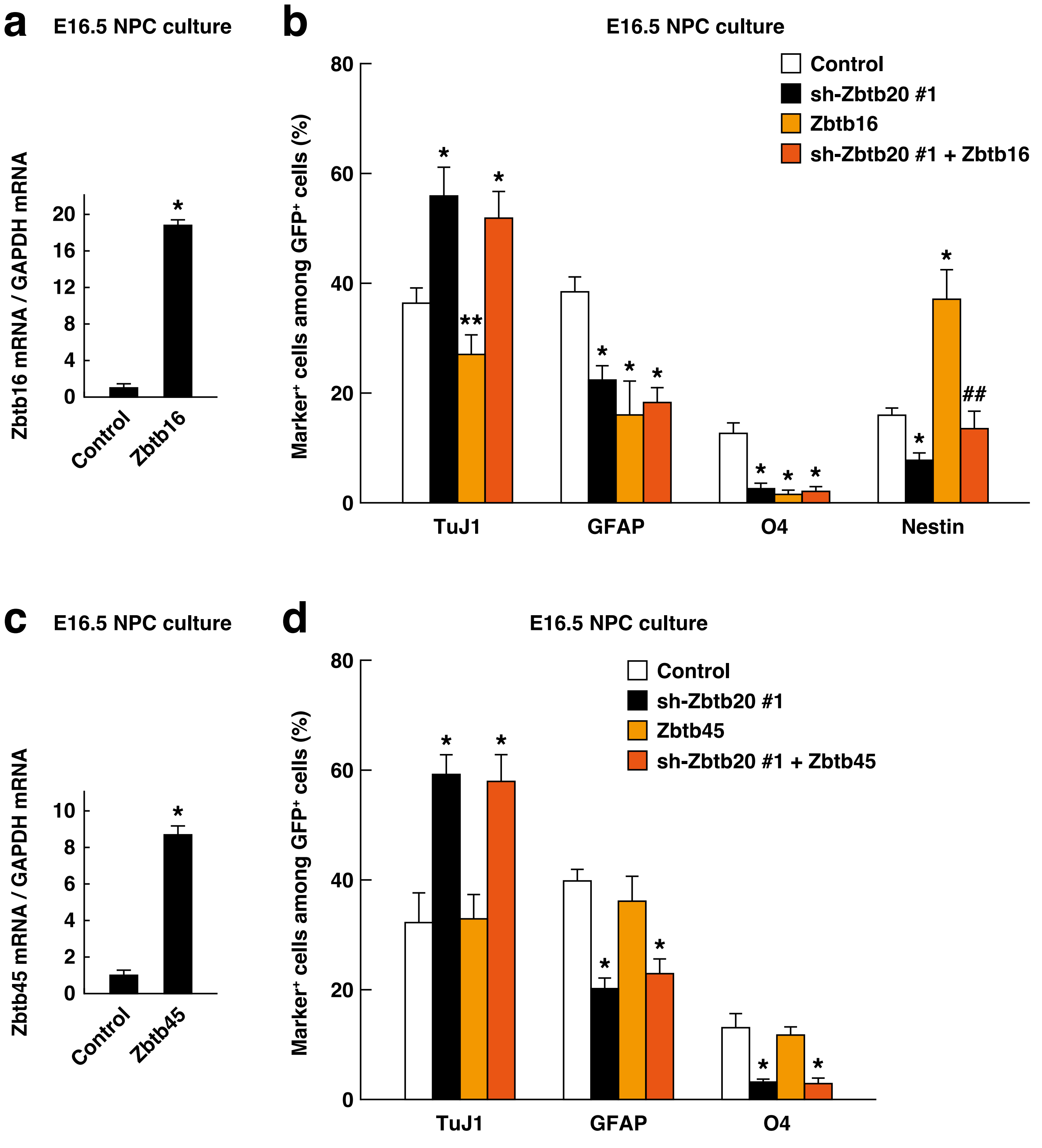

Supplementary Figure 16. Zbtb16 and Zbtb45 fail to rescue the Zbtb20 knockdown phenotypes in NPC cultures. (**a, c**) E16.5 NPCs were infected with retroviruses encoding GFP alone (control) or GFP plus either Zbtb16 (**a**) or Zbtb45 (**c**). The expression level of Zbtb16 (**a**) or Zbtb45 (**c**) mRNA was determined by quantitative RT-PCR analysis. Data are expressed relative to the control value and are means ± s.d. (n = 3). (**b, d**) E16.5 NPCs were infected with retroviruses for control, sh-Zbtb20 #1, Zbtb16, or sh-Zbtb20 #1 plus Zbtb16 (**b**), or for control, sh-Zbtb20 #1, Zbtb45, or sh-Zbtb20 #1 plus Zbtb45 (**d**). The cells were induced to differentiate for 6 days, after which the cells were immunostained for TuJ1, GFAP, O4, nestin, and GFP. The percentages of marker$^+$ cells among total GFP$^+$ cells were quantified as means ± s.d. (n = 3). *P < 0.01, **P < 0.05 versus corresponding control value; ##P < 0.05 versus value for sh-Zbtb20 #1 alone.
